# Investigations into the basal neural-like properties of dental pulp stem cells reveal they possess a functional type 2 muscarinic receptor which regulates quiescence

**DOI:** 10.1186/s13287-025-04730-7

**Published:** 2025-11-12

**Authors:** S. Alqahtani, A. Gibbs, M. Verma, O. Baradwan, K. Jubiar, M. A. Alonazi, W. McLean, C. J. Nile

**Affiliations:** 1 Department of Restorative Dental Sciences, College of Dentistry, Jouf University, Al-Jouf, Saudi Arabia; 2 School of Dental Sciences, Newcastle University, Framlington Place, Newcastle upon Tyne, NE2 4BW UK; 3 Oral Sciences Research Group, University of Glasgow, 378 Sauchiehall Street, Glasgow, G2 3JZ UK

**Keywords:** Dental pulp stem cells, Neurotransmitters, Cholinergic receptors, Acetylcholine, Muscarinic type 2 receptor

## Abstract

**Background:**

Dental pulp stem cells (DPSCs) are a population of mesenchyme-derived cells residing within the dental pulp known for their multipotent differentiation potential and neural-like properties. While a functional cholinergic system has been described in various mesenchymal stem cell (MSC) populations, muscarinic receptor mediated cholinergic signalling remains unexplored in DPSCs.

**Methods:**

The expression of neurotransmitter-associated genes was investigated using a targeted array panel and immunocytochemistry. Functionality of acetylcholine receptors (AChRs) was confirmed using receptor-specific agonists and antagonists. The effects of type 2 muscarinic receptor (m2AChR) signalling on DPSCs viability and proliferation were evaluated using an LDH release assay, CCK-8 assay and annexin V/PI staining. The effect of m2AChR signalling on the cell cycle was determined by flow cytometry and gene expression profiling, and the downstream effects on DPSCs osteogenic differentiation and migration were determined using an osteogenic differentiation assay and a wound healing assay. Finally, the effect of m2AChR signalling on the transcriptome was determined by RNAseq and the role of the MAPK/ERK pathway in mediating m2AChR signalling determined using an in-cell ELISA.

**Results:**

Analysis of the neurotransmitter profile of DPSCs revealed they have cholinoceptive properties and pharmacological investigations confirmed they express a functional m2AChR. Activation of the m2AChR led to a reversible reduction in DPSCs proliferation, without compromising cell viability or pluripotency. Flow cytometric analysis and gene expression profiling confirmed that activation of the m2AChR caused cell cycle arrest at the G2/M phase which coincided with upregulated expression of *CDKN1A* (P21), a canonical marker of quiescence. Activation of the DPSC m2AChR also impaired their migration and osteogenic differentiation capabilities. RNAseq analysis revealed differentially expressed genes involved in regulating the cell cycle and MAPK/ERK signalling. Furthermore, analysis of ERK1/2 phosphorylation suggested that the MAPK/ERK pathway may play a role in m2AChR mediated regulation of quiescence.

**Conclusions:**

DPSCs exhibit cholinoceptive properties, and activation of the m2AChR engages the MAPK/ERK pathway and is associated with a reversible cell-cycle arrest consistent with a quiescent-like state, without affecting viability or pluripotency. These data support the m2AChR as a putative target for manipulating DPSC behaviour and transient quiescence in future regenerative applications.

**Supplementary Information:**

The online version contains supplementary material available at 10.1186/s13287-025-04730-7.

## Introduction

A variety of non-neuronal cells exhibit cholinergic and cholinoceptive properties and are known to express acetylcholine receptors (AChRs) [1, 2]. Cholinergic signalling is hypothesised to occur in stem cells, and there is evidence that mesenchymal stem cells (MSCs) express functional cholinergic systems [3]. Indeed, expression of functional AChRs in several MSC populations suggests that these receptors may contribute to their regenerative potential [3].

Dental pulp stem cells (DPSCs) are derived from mesodermal tissues and are closely related to MSCs. Therefore, it is plausible to speculate that they also exhibit cholinoceptive properties. DPSCs possess membrane properties similar to other neural crest-derived cells [4], including the expression of voltage-gated sodium channels, and have a predisposition for neurogenesis. As such, they have been widely investigated for their potential use in regenerative medicine as an autologous transplantable source of neurons and glial cells to treat neurodegenerative diseases [5, 6]. Furthermore, the neural-like phenotype of DPSCs continues to prompt interest in their basal neurobiological characteristics.

Previous studies have suggested that DPSCs express several neurotransmitter receptors. DPSCs have been suggested to possess a dual dopaminergic and serotonergic identity, expressing both 5-hydroxytryptamine (5-HT) and dopamine receptors [7]. Additionally, they have been shown to express GABA receptors (e.g., GABA receptor α2, β2, and β3) [8] as well as all adenosine receptor subtypes (A1, A2A, A2B, and A3) [9]. Nonetheless, a comprehensive understanding of their basal cholinergic and cholinoceptive properties is lacking.

Cholinergic signalling is mediated by two major classes of membranebound receptors: muscarinic receptors (mAChRs) and nicotinic receptors (nAChRs). Both receptor subtypes are known to be expressed and functional in non-neuronal cells [10], facilitating cell-to-cell communication by activation of intracellular signal-transduction cascades that modulate cellular function and contribute to tissue homeostasis [2].

The nAChRs are ligand-gated ion channels that mediate the ionotropic effects of ACh and are composed of five subunits, arranged as either homo-pentamers or hetero-pentamers [11, 12]. In humans, there are 10 α-subunits (α1 – α10) and four β-subunits (β1–4), along with delta (δ), epsilon (ε), and gamma (γ) subunits [13, 14]. The specific combination of subunits confers unique receptor properties and functional diversity [12].

Muscarinic AChRs are G-protein-coupled receptors (GPCRs) that mediate the metabotropic responses to ACh. In humans there are 5 known subtypes (m1–m5) [15]. Upon activation, these receptors couple with distinct G proteins, triggering various second messenger pathways and ion channel modulation [16]. The metabotropic nature of mAChRs results in slower signalling compared to the ionotropic responses of nAChRs [15]. mAChRs are involved in ACh-mediated effects at nearly all stages of development, and dysregulation of their signalling has been implicated in numerous pathologies [17].

To date, only one study has identified acetylcholine receptor (AChR) expression in DPSCs. Both stem cells from human exfoliated deciduous teeth (SHED) and adult DPSCs were reported to express the α7nAChR, which was implicated in the modulation of osteoclastogenesis and root resorption in deciduous teeth [18]. However, a comprehensive investigation into the broader cholinergic and cholinoceptive properties of DPSCs remains lacking.

## Materials and methods

### Culture of primary human dental pulp stem cells

Primary human dental pulp stem cells (DPSCs) (Lonza Inc, UK) were cultured at 37 °C with 5% CO_2_ in Knock-out Dulbecco’s modified Eagle’s medium (DMEM-KO) (Gibco, UK) supplemented with 10% Foetal bovine serum, 1% L-Glutamine (Sigma, UK‎), and 1% Penicillin-Streptomycin (Gibco, UK). Media was changed every 3 days. When approximately 90% confluent, cells were passaged by detaching with 0.025% trypsin–EDTA (Gibco, UK). All DPSCs used in this study were between passages 4 and 6.

### Human neurotransmitter gene array panel

Applied Biosystems^™^ TaqMan^™^ Array human neurotransmitter plates (Thermo Fisher Scientific, UK) were used to determine expression levels of neurotransmitter associated genes. DPSCs were seeded at 1 × 10^5^ cells per well in 24 well plates and incubated overnight at 37 °C with 5% CO_2_. RNA extraction was performed using an RNAqueous™ Total RNA Isolation Kit (Thermo Fisher Scientific, UK), as per the manufacturer’s instructions. Extracted RNA was quantified and assessed for purity using a Qbit™ 4 (Thermo Fisher Scientific, UK). Extracted RNA was converted to cDNA using an Applied Biosystems™ High-Capacity cDNA Reverse Transcription Kit (Thermo Fisher Scientific, UK). For each plate, a final cDNA concentration of 2 ng per 20 µL reaction was added to the TaqMan^™^ Fast Advanced Master Mix. The plate was loaded into an Applied Biosystems™ QuantStudio 3 Real-Time PCR System. The thermal cycling protocol was as follows: 95 °C for 10 min then 40 cycles of 95 °C for 15 s, and 60 °C for 1 min. To convert Cq values into gene expression levels, the mean Cq value of the 4 reference genes was subtracted from each target gene and a e-ΔCT generated [19].

### Targeted qPCR analysis of cholinergic receptor gene expression

DPSCs were seeded at 1 × 10^5^ cells per well in 24 well plates and incubated overnight at 37 °C with 5% CO_2_. Total RNA was isolated using a RNeasy Mini Kit (Qiagen Ltd, UK) with on column DNase treatment (Qiagen Ltd, UK), according to manufacturer’s protocols. cDNA synthesis was performed using the High-Capacity cDNA Reverse Transcription Kit (Applied Biosystems, UK) according to the manufacturer’s instructions.

Quantitative polymerase chain reaction (qPCR) was performed using a StepOnePlus™ Real-Time PCR System (Applied Biosystems, UK) under the following conditions: 2 min at 95 °C, followed by 40 cycles at 95 °C for 5 s and 60 °C for 30 s. Each sample was analysed in duplicate, and expression of the genes of interest were normalised to the housekeeping gene, *GAPDH*. Relative abundance of a gene transcript was calculated using the e-ΔCT method [19].

### Immunocytochemistry

DPSCs were plated on glass coverslips at a seeding density of 1 × 10^5^ cells per well in DMEM-KO and allowed to adhere overnight. After 24 h, cells were washed with phosphate-buffered saline (PBS) twice and fixed with 4% paraformaldehyde in PBS for 15 min. The cells were washed thrice in PBS with 0.1% Tween 20 (TPBS), permeabilized with 0.1% Triton™ X-100 for 15 min and incubated in TPBS solution containing 1% Bovine Serum Albumin (BSA) for 1 h. The cells were then incubated overnight at 4 °C with primary antibodies targeting the m2AChR (rabbit monoclonal, 1:1000), m3AChR (rabbit polyclonal, 1:1000), or m5AChR (rabbit polyclonal, 1:1000), all diluted in 1% BSA in PBS (Abcam, UK). The next day, the cells were washed with TPBS and then incubated with a goat anti-rabbit secondary antibody (1:1000, Goat anti-Rabbit IgG (H + L) Alexa Fluor™ 488) (Invitrogen, UK) diluted in 1% BSA in PBS for 1 h in the dark at RT. After three washes in TPBS, cells were then stained with Alexa Fluor 488 phalloidin (Invitrogen, UK) for 30 min to label actin filaments. After 3 washes in TPBS, coverslips were mounted with Vectashield Antifade mounting medium containing 4’,6-diamidino-2-phenylindole (DAPI) (Fisher Scientific, UK). Images were captured using an EVOS FL digital inverted microscope (Thermo Fisher Scientific, UK) equipped with a monochrome camera and a 40x phase objective. Image merges were created by the built-in microscope software.

### Pharmacological determination of functional cholinergic receptor expression

To assess functionality of detected AChRs, the metabolic activity of DPSCs was determined in the presence and absence of the following cholinergic receptor agonists and antagonists; (i) m1AChR > m2AChR, Agonist = 100 µM McN-A 343 (Sigma Aldrich, UK), Antagonist = 0.1 µM Pirenzepine (Sigma Aldrich, UK), (ii) m2AChR, Agonist = 100 µM Arecaidine propargyl ester hydrobromide (APE) (Sigma Aldrich, UK), Antagonist = 0.1 µM Methoctramine (Sigma Aldrich, UK).

Briefly, cells were seeded into 96-well plates at a density of 1 × 10^4^ cells/well in DMEM-KO. After 24 h, cells were treated with subtype specific mAChRs agonists and antagonists. Pharmacological competitions were performed by pre-treating cells with antagonists 2 h prior to agonist exposure, based on previously published protocols [20, 21] After 72 h, cell metabolic activity was assessed using a 3‐(4,5‐dimethyl thiazol 2‐y1) ‐2,5‐diphenyl tetrazolium bromide (MTT) assay (Sigma Aldrich, UK). For each well, media was replaced with 100 µL MTT solution (0.5 mg/ml) and incubated for 4 h at 37 °C and 5% CO_2_. Subsequently, the dye was replaced with 100 µL of dimethyl sulfoxide (DMSO) and incubated for 1 h at 37 °C and 5% CO_2_. Optical density readings were performed on a FLUOstar Omega microplate reader (Thermo Fisher Scientific, UK) at 545 nm and 650 nm.

For recovery experiments, the m2AChR selective agonist APE (100 µM) was first used to stimulate the receptor and MTT measurements taken between 24 and 144 h. Next, the experiment was repeated, but after 72 h of APE treatment the media was removed and the cells washed thrice with PBS prior to replacing with fresh growth media without APE and MTT assays performed over the next 72 h. A concentration of 100 µM APE was initially selected based on previous studies [20–23].

### LDH assay

To determine DPSC plasma membrane damage after treatment with 100 µM APE, levels of released Lactate dehydrogenase (LDH) were measured using a LDH cytotoxicity assay kit (Thermo Fisher Scientific, UK) according to manufacturer’s instructions. Briefly, DPSCs were plated into 96-well plates at a density of 1 × 10^4^ cells/well and incubated with 100 µM APE for 72 h. Cells lysed with lysis buffer acted as a positive control and cells in media alone acted as a negative control. At the end of the treatment, 20 µL of cell supernatant was mixed with 20 µL of the LDH reaction mix and incubated in the dark for 30 min at room temperature (RT). After incubation, the reaction was stopped by adding 20 µL of stop solution. Absorbance was measured at 490 nm with a reference wavelength of 680 nm on a FLUOstar Omega microplate reader (Thermo Fisher Scientific, UK). Percentage cytotoxicity was determined as per the manufacturer’s instructions.

### CCK-8 assay

To evaluate LDH levels in viable cells after treatment with 100 µM APE, a CCK-8 cell counting kit (Dojindo, Japan) was employed. Briefly, DPSCs were plated into 96-well plates at a density of 1 × 10^4^ cells/well and incubated with 100 µM APE for up to 120 h. Serum-starved cells served as negative controls, while cells cultured in complete medium served as positive controls. At various timepoints, 10 µL of the assay solution was added to each well of the plate and the plate incubated for 4 h at 37 °C with 5% CO_2_. Subsequently, absorbance was measured at 450 nm on a FLUOstar Omega microplate reader (Thermo Fisher Scientific, UK).

### Annexin V/PI staining

To determine whether APE induced DPSC necrosis or apoptosis, an Annexin V-fluorescein isothiocyanate (FITC)/propidium iodide detection kit (Abcam, UK) was employed. The experiment was performed according to the manufacturer’s protocol. Briefly, DPSCs were plated into 24-well plates at a density of 5 × 10^4^ cells/well and incubated with 100 µM APE for 72 h. Positive controls to detect PI and Annexin V staining were generated using 30% methanol as an inducer of apoptosis, and 70% methanol as an inducer of necrosis. Cells were sequentially incubated with 5 µL propidium iodide (PI) binding buffer (50 µg/ml) and 5 µL Annexin V-FITC (10 µg/ml) in the dark. Subsequently, cells were fixed with 2% paraformaldehyde in PBS for 15 min at RT, washed, and stained with DAPI mounting medium. Images were captured using an EVOS FL digital inverted microscope (Thermo Fisher Scientific, UK) equipped with a monochrome camera and a 40x phase objective. Images for two-channel merges were created by the built-in microscope software.

### Cell cycle analysis

DPSCs were plated into a 75-cm^2^ flasks at a seeding density of 1.5 × 10^6^ and allowed to adhere overnight. The following day, cells were treated with 100 µM APE for 72 h. To enable analysis of cells in the S phase, cells were incubated with 50 µM bromodeoxyuridine (BrdU) (Sigma Aldrich, UK) after 48 h stimulation with 100 µM APE. After 72 h, cells were harvested by trypsinisation, centrifuged for 5 min at 1200 rpm and then fixed in 70% ethanol overnight. Partial DNA denaturation was performed by incubating the cells in 2 N HCl for 30 min at 37 °C, followed by neutralization with 0.1 M sodium tetraborate. Cells were spun out of neutralised acid and washed with PBS containing 2% FBS (PBS-F). Cells were then incubated overnight at 4 °C with anti-BrdU clone MoBU-1 conjugated to Alexa Fluor™ 488 (Invitrogen, UK) in PBS-F. The following day, cells were incubated in a propidium iodide/RNase staining buffer (BD biosciences, UK) for 30 min at RT. Flow cytometry analysis was performed on a MACSQuant Analyzer 10 Flow Cytometer (Miltenyi Biotech, UK) with 488 nm wavelength excitation, and 100 events were collected for each sample. Analyses were performed using FLOWJO V.10 software (Treestar Inc., OR, USA) in which curve fitting was used to determine the percentages of cells in each phase of the cell cycle.

### Cell migration assay

To determine the effect of m2AChR activation on DPSCs migration, a wound healing assays was performed [24]. Cells were plated on a 6-well plate at a seeding density of 2 × 10^5^ cells per well. The following day, cells were treated with 100 µM APE, with or without 0.01 µM Methoctramine, and a scratch was made using a 200 µL pipette tip (Starlab, UK). Mitomycin C (50 ng/mL, Fisher BioReagents, UK) was added to the medium of treated and untreated cells to exclude the possible influence of cell proliferation. The cells were photographed using an EVOS FL digital inverted microscope (ThermoFisher Scientific, UK) at 0 h and after 8 h. The cells were then stained with phalloidin (Alexa Fluor™ 488, Invitrogen, UK) for 30 min at RT to label actin filaments. The space between the two cell fronts at 0 h and 8 h was then measured using ImageJ (64-bit) imaging software [25]. And the two values subtracted to determine the width of the scratch.

### Assessment of stemness and ability for osteogenic differentiation

To determine whether m2AChR activation affects DPSC stemness, expression of the genes encoding CD90, CD105, CD73 and CD45 were evaluated after stimulation with 100 µM APE for 72 h. Furthermore, expression of genes encoding the transcription factors Oct4, Nanog and Sox-2 were also evaluated due to their role in maintenance of self-renewal and pluripotency.

The effect of m2AChR activation on the differentiation capabilities of DPSCs was determined using an osteogenic differentiation assay. Briefly, cells were seeded in 24-well plates and cultured in complete culture medium at a seeding density of 1 × 10^4^ cells per well. When cells reached 85–90% confluence (48–72 h), medium was changed to osteogenic inductive medium (100 nM dexamethasone, 50 µM ascorbic acid-2-phosphate, and 10 mM β-glycerophosphate). At onset, and then at subsequent time points, 100 µM APE was added to the osteogenic inductive medium to observe its effect on, and during, the differentiation process. Cells were incubated at 37 °C with 5% CO_2_ for 7, 14, 21, and 28 days, and media was refreshed every 3 days. At each time point, mineralisation was assessed by Alizarin Red (40 mmol/L, pH 4.2) (Sigma–Aldrich, UK) and Von Kossa (Sigma–Aldrich, UK) staining. In addition, total RNA was harvested for evaluation of expression of genes involved in osteogenesis.

### RNA sequencing

DPSCs (5 × 10^5^) were incubated with 100 µM APE for 4 and 24 h. Total RNA was then isolated using a RNeasy Mini Kit (Qiagen Ltd, UK) with on column DNase treatment (Qiagen Ltd, UK), according to manufacturer’s protocols. The quality and quantity of RNA was determined using a Bioanalyzer 2100 (Agilent Technologies, CA, USA) and samples with a minimum RNA integrity number (RIN) of 7.0 and a minimum quantity of ≥ 20 ng/µL were sent for sequencing by Novogene (Novogene Co LTD, Cambridge, UK). RNA sequencing and bioinformatics analysis was performed using in-house Perl scripts, following the workflow shown in Supplementary Fig. 1. The data discussed in this publication have been deposited in NCBI’s Gene Expression Omnibus and are accessible through GEO Series accession number GSE297021 (https://www.ncbi.nlm.nih.gov/geo/query/acc.cgi? acc=GSE297021).

### ERK1/2 Enzyme-linked immunosorbent assay

To evaluate the activation of the extracellular signal-regulated kinases (ERK) 1 & 2, a Phospho-ERK1 (T202/Y204) / ERK2 (T185/Y187) incell ELISA (R&D Systems, UK) was performed. Briefly, 1 × 10^4^ DPSCs were incubated with 100 µM APE for 0–40 min at 37 °C. The cells were washed twice with PBS, lysed and incubated on ice for 15 min. Samples were then centrifuged at 2000 x g for 5 min and the supernatant isolated. A Bicinchoninic acid assay (Thermo Scientific, UK) was carried out to standardise protein concentration. The capture antibody was incubated in a 96-well plate overnight at room temperature (RT). The following day, the plate was washed three times with wash buffer. The plate was then incubated in blocking buffer for 1 h at RT, washed three times, and the Phospho-ERK1/ERK2 standard and samples were loaded onto the plate. The plate was then incubated for 2 h at RT, washed and the detection antibody added. After further incubation for 2 h at RT the plate was again washed and incubated with streptavidin-HRP for 20 min at RT. After washing again, substrate solution (1:1, H_2_O_2_: tetramethylbenzidine) was loaded onto the plate which was then incubated for 20 min at RT in the dark. Finally, stop solution (2 N sulphuric acid) was added to each well, and the plate was read on a microplate reader (FLUOstar Omega microplate reader) at 450 nm and 540 nm. The concentrations of phospho-ERK1/2 in the cell lysates were determined from a standard curve and differences between untreated and treated cells determined.

### Statistical analysis

Statistical analysis was performed with GraphPad Prism for macOS (GraphPad Software, Inc., La Jolla, CA, USA). Normal distribution of the investigated samples was assessed using Shapiro-Wilk normality test. Analysis of two conditions was done with an unpaired t test followed by a Mann-Whitney test. Analysis of more than two conditions were done by one-way ANOVA with Dunnett’s multiple comparison, Tukey’s post hoc or Kruskal-Wallis tests. The data are presented as mean ± Standard Error of the Mean (SEM) with a difference of *P* < 0.05 considered statistically significant. Differential gene expression analysis of the RNA sequencing data is presented as p adjusted values (padj) using the Benjamini and Hochberg’s approach for controlling the false discovery rate. Genes with a padj ≤ 0.05 found by DESeq2 were considered statistically significant.

## Results

### Analysis of the basal neural-like properties of dental pulp stem cells reveals cholinoceptive capabilities

**Fig. 1:**
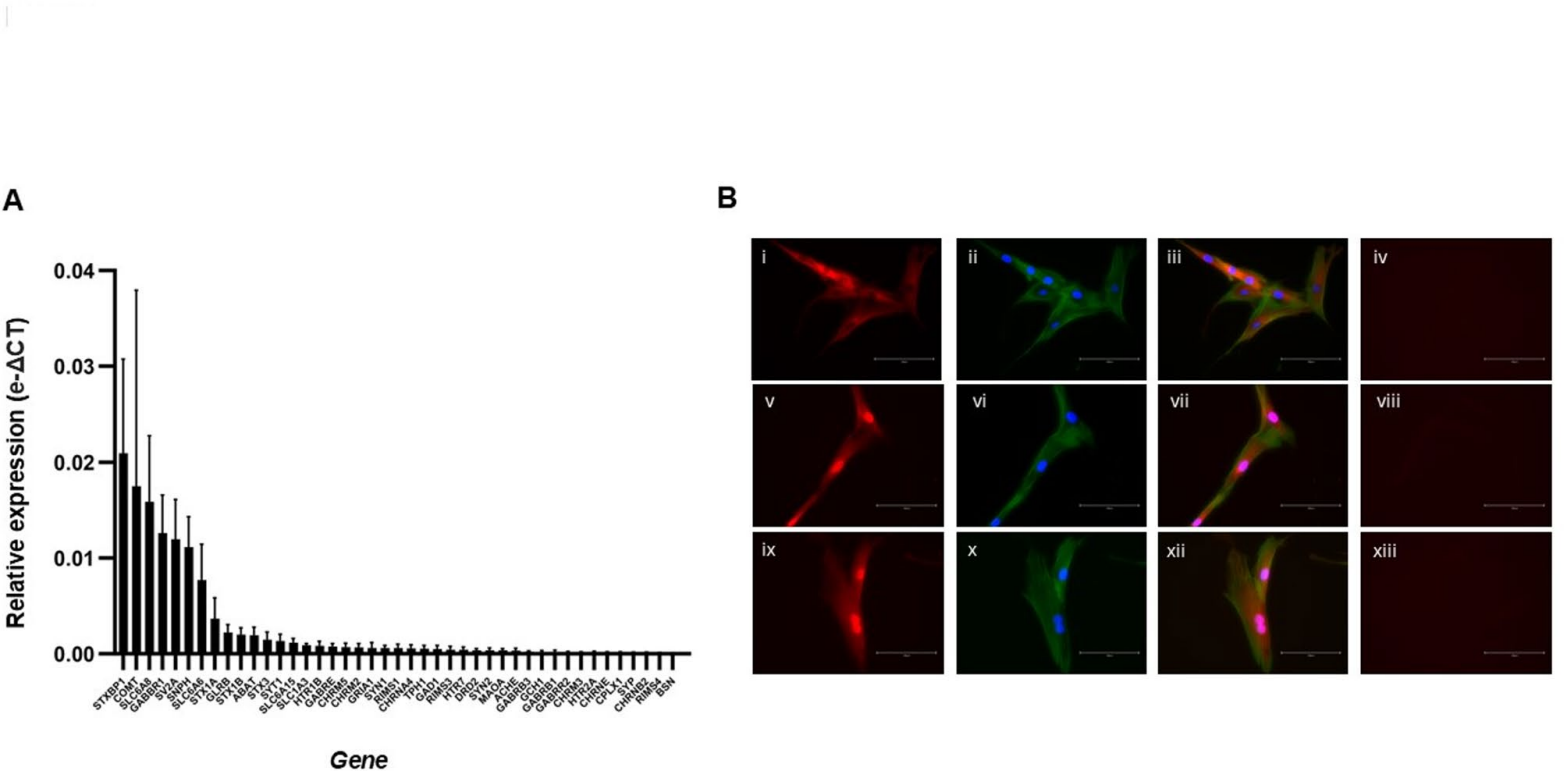
Analysis of the dental pulp stem cell neurotransmitter profile reveals expression of muscarinic receptors. **A** Mean gene expression values for the 43/92 neurotransmitter genes determined to be expressed in DPSCs calculated using the e-ΔCT method. The data are derived from six biological replicates (*n* = 6). **B** Immunocytochemical analysis of m2AChR, m3AChR and m5AChR expression by DPSCs. **(i)** Expression of the m2AChR protein in DPSCs, **(ii)** Merged images showing DPSCs nuclei stained with DAPI (blue) and actin filaments with phalloidin (green), **(iii)** Merged images of **(i)** and **(ii)**, and **(iv)** Negative control with no primary antibody showing no non-specific binding. **(v)** Expression of the m3AChR protein in DPSCs, **(vi)** Merged images showing DPSCs nuclei stained with DAPI (blue) and actin filaments with phalloidin (green), **(vii)** Merged images of **(v)** and **(vi)**, and **(viii)** Negative control with no primary antibody showing no non-specific binding. **(ix)** Expression of the m5AChR protein in DPSCs, **(x)** Merged images showing DPSCs nuclei stained with DAPI (blue) and actin filaments with phalloidin (green), **(xi)** Merged images of **(ix)** and **(x)**, and **(xii)** Negative control with no primary antibody showing no non-specific binding. All images show 2D projections of confocal stacks and are representative of three independent experiments (*n* = 3). Scale bars = 100 μm

Expression levels of neurotransmitter associated genes in DPSCs were determined using Applied Biosystems^™^ TaqMan^™^ Array human neurotransmitter plates. Figure 1A shows that 43 of the 92 neurotransmitter genes on the panel were expressed in DPSCs. The most relatively highly expressed genes were *STXBP1* (syntaxin binding protein-1), *COMT* (Catechol-O-Methyltransferase), *SLC6A8* (sodium-and chloride dependent creatine transporter-1), *GABBR1* (Gamma-Aminobutyric Acid Type B Receptor Subunit-1) and *SV2A* (Synaptic Vesicle Glycoprotein 2 A).

Several genes expressed by DPSCs were found to be involved in the metabolism and response to acetylcholine (supplementary Fig. 2A). To further interrogate whether DPSCs express cholinergic components, targeted qPCR was employed. DPSCs were found to express, gene transcripts for the *CHRNA4* (α4), *CHRNA7* (α7), *CHRNA9* (α9), *CHRNB1* (β1), *CHRNB2* (β2), and *CHRNE* (ε) nicotinic receptor subunits, gene transcripts for *CHRM2* (m2AChR), *CHRM3* (m3AChR), and *CHRM5* (m5AChR) and gene transcripts for acetylcholinesterase (*ACHE*) (supplementary Fig. 2B – 2D).

To confirm whether expression of gene transcripts for the m2AChR, m3AChR and m5AChR translated to expression of the relevant proteins, immunocytochemistry was performed. Positive immunostaining for the m2AChR, m3AChR and m5AChR was detected in DPSCs (Fig. 1B).

### Dental pulp stem cells express a functional muscarinic type 2 receptor and activation induces a reversible inhibition of cell proliferation

**Fig. 2:**
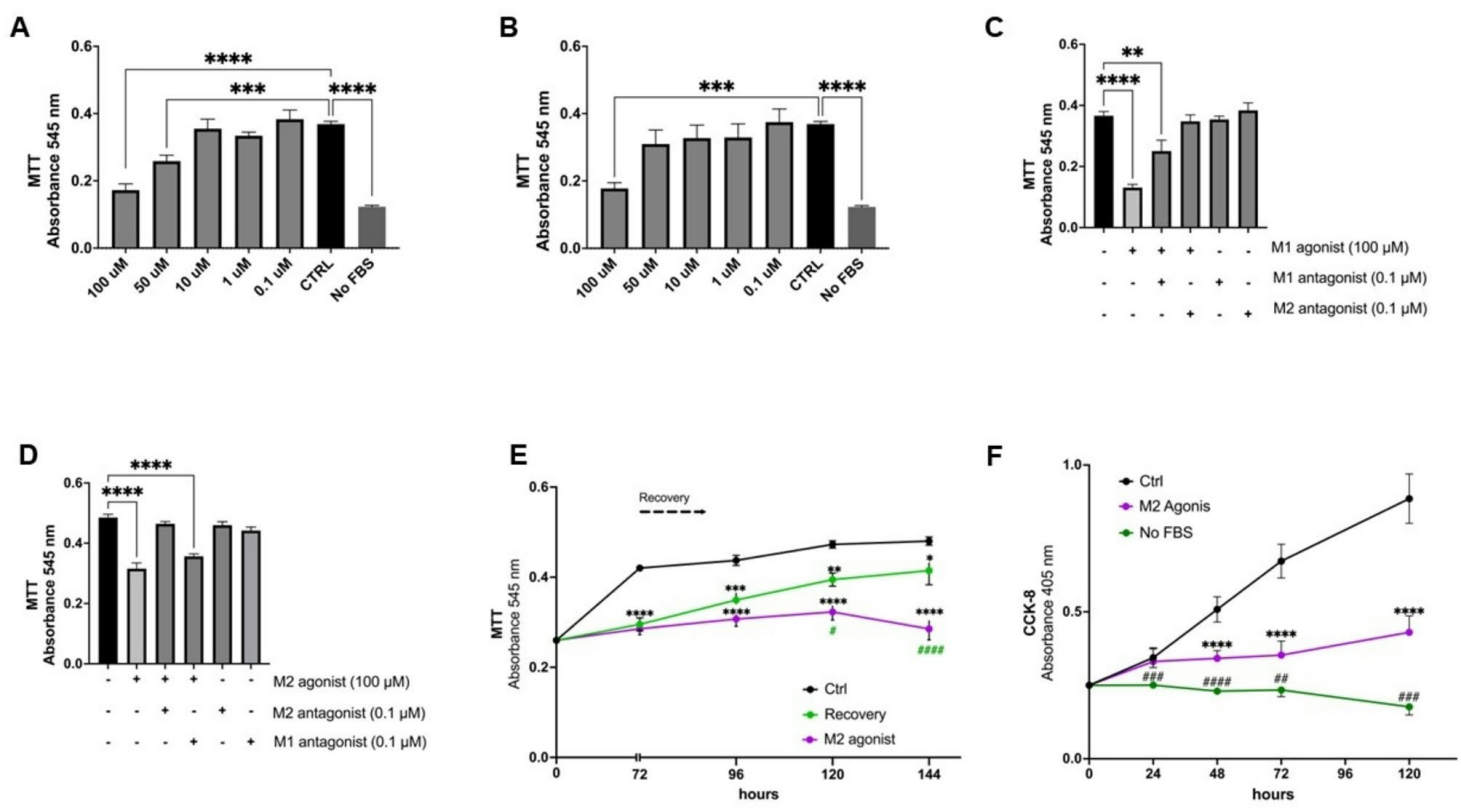
Pharmacological induction and inhibition of dental pulp stem cell proliferation confirm the expression of a functional m2AChR. The effect of the m2AChR agonists **A** McN-A 343 (McN, 0.1–100 µM) and **B** Arecaidine propargyl ester hydrobromide (APE, 0.1–100 µM) on DPSC proliferation determined using an MTT assay. The effects on DPSC proliferation after 2 h pretreatment with 100 µM Pirenzepine (PZ) and 0.1 µM Methoctramine (MC), alone or in combination, followed by 72 h treatment with **C** 100 µM McN and **D** 100 µM APE. Comparison made to untreated group (CTRL); negative controls are cells without serum (No FBS). **E** Time course of DPSC proliferation after stimulation with 100 µM APE. Ctrl denotes DPSCs cultured in media alone, 100 µM denotes DPSCs cultured in media containing 100 µM APE and recovery denotes DPSCs cultured in media containing 100 µM APE for 72 h and then replacing with culture media alone. **F** CCK-8 assessment of live cells over a 120 h period. m2AChR agonist depicts viable cells after stimulation with 100 µM APE. Serum starved cells (No FBS) acted as a negative control and cells in media alone containing serum (Ctrl) acted as a positive control. Data are presented as mean ± SEM from duplicate wells across three independent experiments. Statistical analysis was performed using one-way ANOVA; Dunnett’s multiple comparisons test was used for panels A–D, and Tukey’s post hoc test was used for panels E and F. *p < 0.05, **p < 0.01, ***p < 0.001, ****p < 0.0001. Full dose–response and recovery data for APE are shown in Supplementary Fig. 3B, and antagonist-alone controls are provided in Supplementary Fig. 3A

To determine if DPSCs expressed a functional m2AChR, proliferation was assessed following treatment with pharmacological agonists and antagonists. DPSCs proliferation was inhibited in the presence of the m2AChR agonists, McN-A 343 (McN), significantly at 50 and 100 µM (Fig. 2A), and Arecaidine propargyl ester hydrobromide (APE), significantly at 100 µM (Fig. 2B; dose–response and recovery data shown in Supplementary Fig. 3B).

To confirm that the effect on DPSCs proliferation was subtype specific, and since McN is reported to have off-target effects on the m1AChR in humans, experiments with McN and APE were repeated in the presence and absence of Pirenzepine (PZ; a selective m1AChR antagonist) and/or Methoctramine (MC, a selective m2AChR antagonist). Neither PZ or MC alone had any effect on DPSC proliferation (supplementary Fig. 3A). Interestingly, MC, but not PZ, abolished the McN (Fig. 2C) and APE (Fig. 2D) induced inhibition of proliferation.

To determine whether activation of the m2AChR led to irreversible changes in DPSCs proliferation capacity, recovery experiments were performed. DPSCs treated with 100 µM APE exhibited significantly inhibited proliferation after 72 h of treatment (*P* < 0.001). However, withdrawal of APE after 72 h of treatment resulted in a resumption of proliferation over the next 72 h suggesting that the inhibitory effect of APE was reversible (Fig. 2E). To confirm that, in line with the reported literature, 100 µM APE was the optimal concentration, dose-response inhibition and recovery experiments were performed using concentrations ranging from 0 to 500 µM. The data suggested that concentrations of APE ≥ 250 µM caused irreversible changes in cell proliferation suggestive of cellular toxicity (supplementary Fig. 3B) and therefore 100 µM APE was used throughout the remainder of this study, in line with previous studies [20–23]. Furthermore, CCK-8 analysis, which measures dehydrogenase levels inside viable cells, showed that DPSCs incubated in 100 µM APE are still viable over the experimental time course, compared to cells deprived of serum. However, there were significantly less cells compared to the control (*P* < 0.0001) indicative of inhibited proliferation (Fig. 2F). Furthermore, an LDH assay and Annexin V/PI staining also confirmed that APE had no cytotoxic effects on DPSCs, including the initiation of apoptosis (supplementary Fig. 4).

### Activation of the dental pulp stem cell muscarinic type 2 receptor arrests cell cycle progression but has no impact on their stemness

**Fig. 3:**
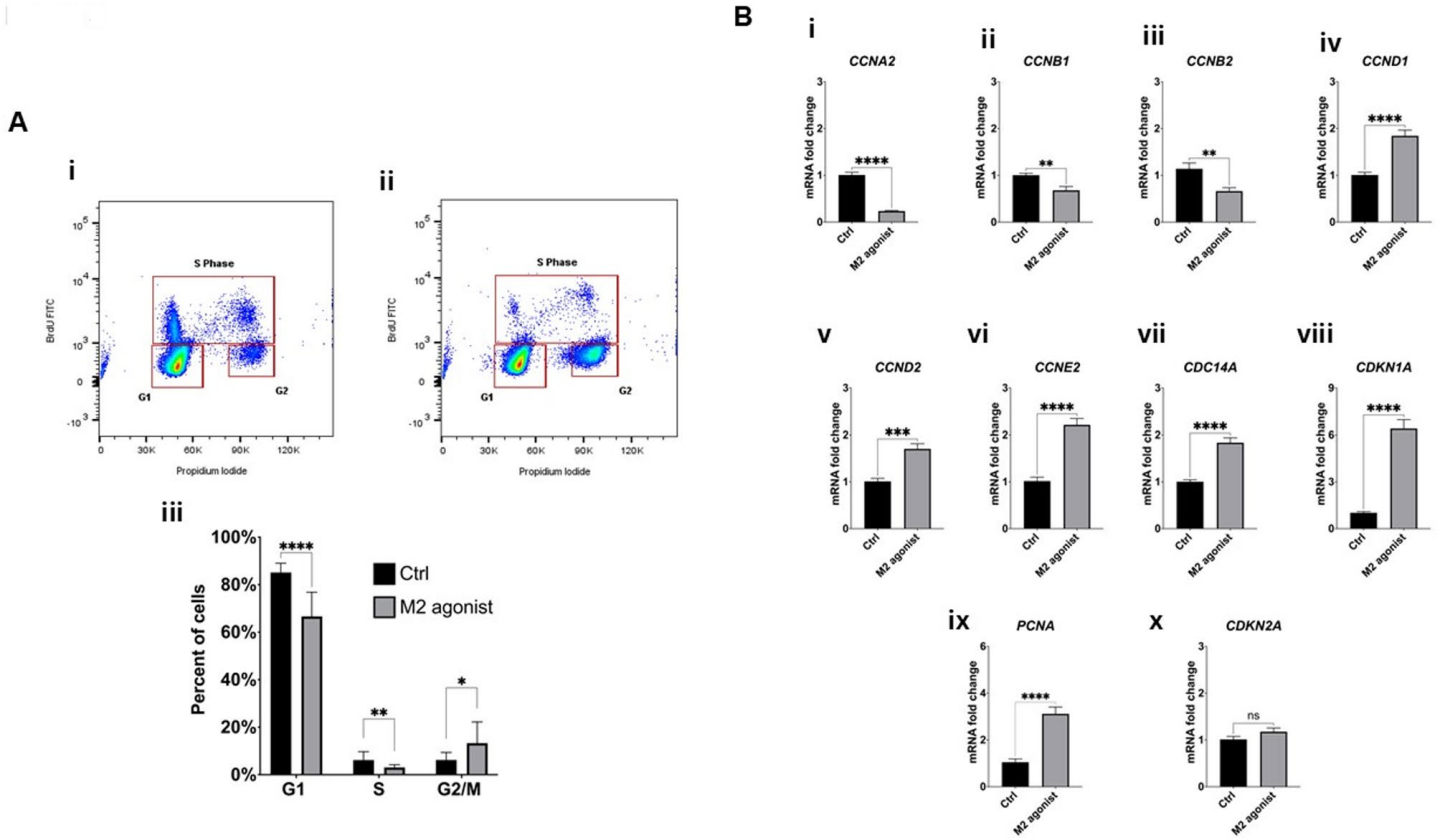
Activation of the m2AChR arrests dental pulp stem cell proliferation at the G2/M phase. **A** Cell cycle analysis of DPSCs after 72 h of stimulation with the M2 agonist (100 µM APE) using bivariate analysis of BrdU incorporation (ordinate) and propidium iodide (PI) labelled DNA content (abscissa). **(i)** Example bivariate plot of DPSCs cultured in media alone (ctrl) and gating strategy. **(ii)** Example bivariate plot of DPSCs cultured in media containing 100 µM APE (M2 agonist) and gating strategy. **(iii)** Graphical representation of cell cycle analysis. **B** Analysis of expression of genes involved in proliferation and the cell cycle following stimulation with the M2 agonist (100 µM APE) for 72 h. **(i)** cyclin-A2 (*CCNA2*), **(ii)** cyclin-B1 (*CCNB1*), **(iii)** cyclin-D1 (*CCND1*), **(iv)** cyclin-D2 (*CCND2*), **(v)** cyclin-E2 (*CCNE2*), **(vi)** cell division cycle 14 A (*CDC14A*), **(vii)** M phase inducer phosphatase 1 (*CDC25A*), **(viii)** cyclin dependent kinase inhibitor 1 A (*CDKN1A*), **(ix)** glycogen synthase kinase 3 beta (*GSK3B*) and **(x)** proliferating cell nuclear antigen (*PCNA*). Cells cultured in media (undifferentiated) alone acted as a control (ctrl). M2 agonist = cells cultured in media containing 100 µM APE. Data are presented as mean ± SEM from duplicate wells across three independent experiments. Statistical analysis was performed using unpaired two-tailed t-tests for all comparisons. *p < 0.05, **p < 0.01, ***p < 0.001, ****p < 0.0001

The evidence suggests that activation of the m2AChR causes reversible inhibition of proliferation without affecting DPSCs viability. This suggested that the m2AChR may modulate cell cycle progression. Cell cycle analysis was performed by BrdU and PI staining. Figure 3A shows a significant reduction of cells in both the G1 ( *P* < 0.0001) and S (*P* < 0.01) phase, and accumulation of cells in the G2/M phase (*P* < 0.0001), after incubation with 100 µM APE. Furthermore, Fig. 3B shows that activation of the m2AChR with 100 µM APE caused a significant downregulation in expression of transcripts encoding cyclin-A2 (*CCNA2*, *P* < 0.0001), cyclin-B1 (*CCNB1*, *P* < 0.01) and cyclin-B2 (*CCNB2*, *P* < 0.01). In contrast, m2AChR activation caused upregulation of cyclin-D1 (*CCND1*, < 0.001), cyclin-D2 (*CCND2*, *P* < 0.0001), cyclin-E2 (*CCNE2*,  *P* < 0.0001), cell division cycle 14 A (*CDC14A*, *P* < 0.0001), cyclin dependent kinase inhibitor 1 A (P21) (*CDKN1A*, *P* < 0.0001) and proliferating cell nuclear antigen (*PCNA*, *P* < 0.0001). However, no difference in expression of cyclin dependent kinase inhibitor 2 A (P16) (*CDKN2A*, ns) was observed.

DPSCs are positive for mesenchymal stem cell stemness markers including CD90, CD105, and CD73. However they do not express CD45 along with several other hematopoietic stem cell markers [26]. Furthermore, DPSCs also express markers of pluripotency such as Sox2, Nanog, and Oct4 [27]. To confirm that activation of the m2AChR did not induce spontaneous differentiation, or affect stemness, expression of these key genes was investigated after 72 h exposure to 100 µM APE. The results demonstrate that APE had no effect on DPSCs expression of CD90 (*THY1*), CD105 (*ENG*), CD73 (*NT5E*), *Sox2*, *Nanog* and *Oct4* and confirmed that they do not express CD45 (*PTPRC*) (supplementary Fig. 5i - vii). These data suggest that activation of the m2AChR with a selective agonist modulates cell cycle progression and places DPSCs in a quiescent state without affecting their stemness or pluripotency.

### Activation of the dental pulp stem cell muscarinic type 2 receptor inhibits their migration and osteogenic differentiation capabilities

**Fig. 4:**
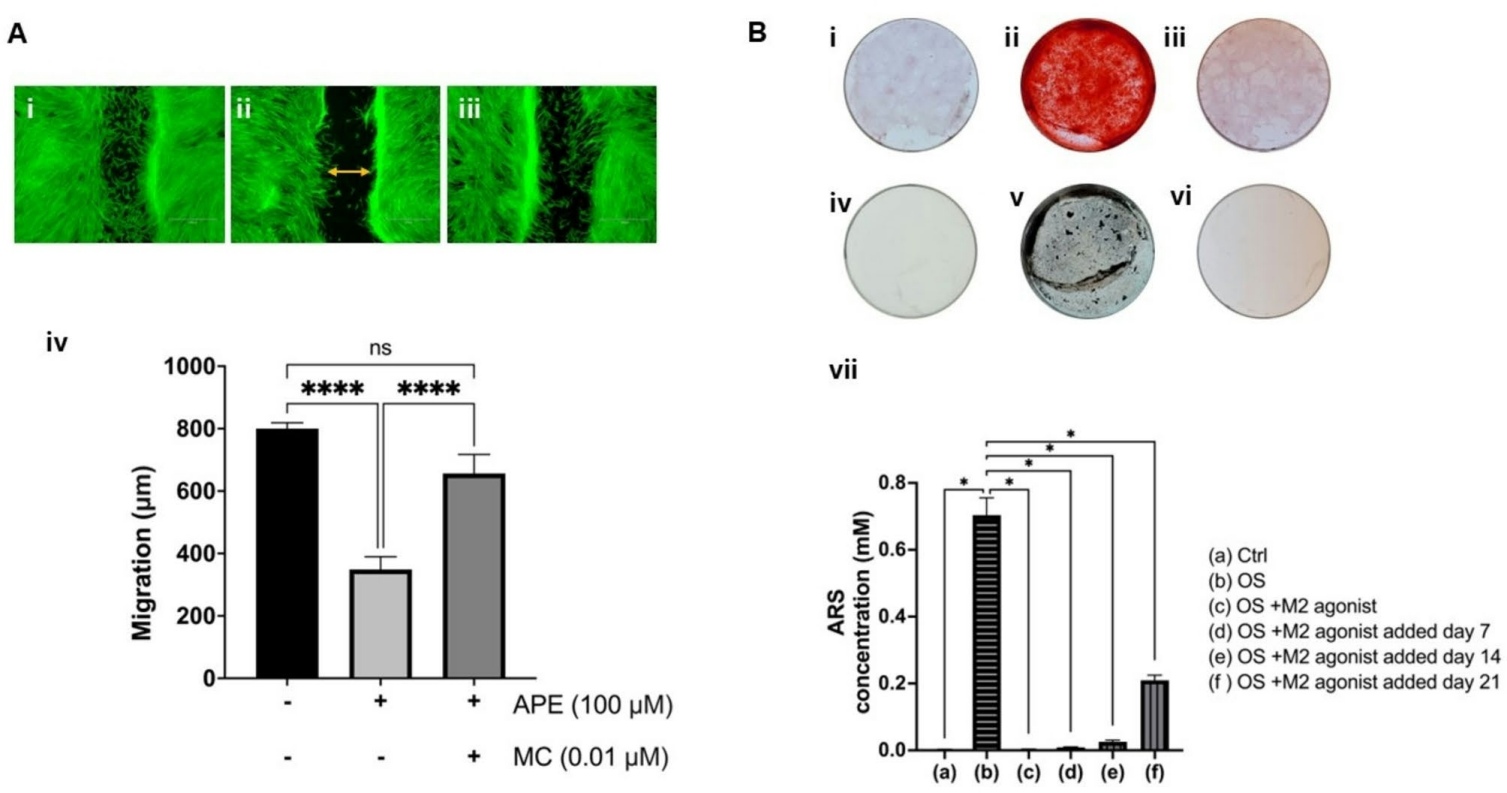
Activation of the dental pulp stem cell m2AChR inhibits their migration and osteogenic differentiation. **A** Determination of the effect of m2AChR activation on DPSC migration using a scratch assay. **(i)** DPSCs grown on glass coverslips in control media, exposed to a scratch and stained for actin (green) after 8 h. **(ii)** DPSCs grown on glass coverslips in media containing 100 µM APE, exposed to a scratch and stained for actin (green) after 8 h. **(iii)** DPSCs grown on glass coverslips in media containing 0.01 µM MC (m2AChR antagonist) and 100 µM APE, exposed to a scratch and stained for actin (green) after 8 h. Images are representative of duplicate coverslips from three independent experiments. **(iv)** Quantification of DPSC migration measured by determining the difference in the width of the gap (as shown by yellow arrow) at 0 h and 8 h. Statistical analysis was performed using one-way ANOVA with Tukey’s post hoc test. Data are presented as mean ± SEM (*n* = 3). ns: not significant, ****p < 0.0001. Scale bars = 1200 μm. APE = Arecaidine propargyl ester, MC = Methoctramine. **B** Determination of the effect of m2AChR activation on DPSC osteogenic differentiation. DPSCs grown on glass coverslips for 4 weeks in normal media and stained with Alizarin red. **(ii)** DPSCs grown on glass coverslips for 4 weeks in osteogenic differentiation media and stained with Alizarin red. **(iii)** DPSCs grown on glass coverslips for 4 weeks in osteogenic differentiation media containing 100 µM APE and stained with Alizarin red. **(iv)** DPSCs grown on glass coverslips for 4 weeks in normal media and stained with von Kossa. **(v)** DPSCs grown on glass coverslips for 4 weeks in osteogenic differentiation media and stained with von Kossa. **(vi)** DPSCs grown on glass coverslips for 4 weeks in osteogenic differentiation media containing 100 µM APE and stained with von Kossa. Images are representative of duplicate coverslips from three independent experiments. **(vii)** Alizarin Red stain quantification showing significantly less mineralisation compared to cells that had undergone osteogenic differentiation. Statistical analysis was performed using one-way ANOVA with Kruskal–Wallis post hoc test. Data are presented as mean ± SEM (*n* = 3). *p < 0.05, ****p < 0.0001

To evaluate the effect of m2AChR activation on DPSCs migration, a wound healing assay was performed. Figure 4A shows that activation of the DPSC m2AChR with 100 µM APE significantly inhibits cell migration (*P* < 0.0001), and this inhibition of migration could be attenuated by pretreatment with the selective m2AChR 0.1 µM of the antagonist MC.

To evaluate the effect of m2AChR activation on DPSCs osteogenic differentiation, DPSCs were incubated for 4 weeks in osteo-inductive medium (OS) with and without 100 µM APE added at various stages during the osteogenic differentiation process. Figure 4B shows that in the presence of OS alone after 4 weeks the cells formed mineralised nodules as confirmed by Alizarin Red and Von Kossa staining. In contrast the formation of mineralised nodules was clearly inhibited in the presence of 100 µM APE. In addition, Alizarin Red stain quantification revealed that addition of 100 µM APE at the start of differentiation and after 7, 14 and 21 days significantly inhibited mineralisation. Furthermore, analysis of expression of key genes involved in the osteogenic differentiation process showed that addition of 100 µM APE inhibited the OS media induced expression of alkaline phosphatase (*ALP*, *P* < 0.0001), bone morphogenic protein 2 (*BMP2*, *P* < 0.001), alpha-1 type I collagen (*COL1A1*, *P* < 0.0001) and secreted phosphoprotein 1 (*SPP1*, *P* < 0.05). In contrast, although there was a small decrease in expression of *RUNX2*, this was not statistically significant (supplementary Fig. 5, viii - xii).

### Activation of the dental pulp stem cell muscarinic type 2 receptor results in differentially expressed genes

Whole transcriptomic sequencing was performed to holistically investigate the effect of activation of the DPSCs m2AChR. All sequenced samples passed the quality control criteria and exhibited > 96% mapping to the reference genome (Human Genome) (data not shown). Multivariate analysis using principal component analysis (PCA) showed that the duration of stimulation caused the highest variance in clustering by 52% (x-axis), followed by 11% due to the nature of the stimulation (supplementary Fig. 6A).

**Fig. 5:**
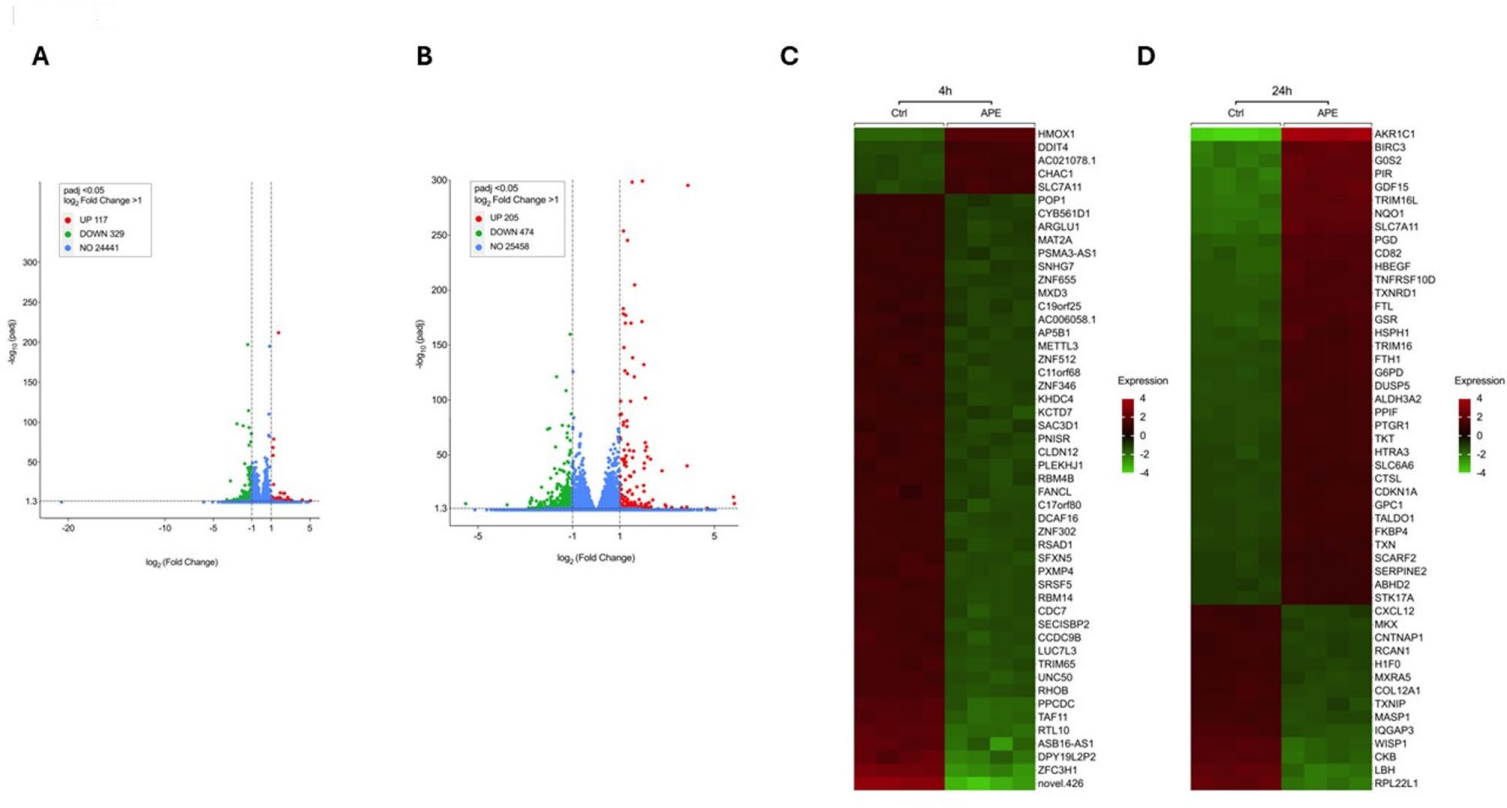
Overview of differential gene expression by dental pulp stem cells after activation of the m2AChR. Volcano plots for differentially expressed genes (DEGs) of DPSCs after activation of the m2AChR for 4 h **(A)** and 24 h **(B)** compared to untreated controls. Scatter points represent genes. The x-axis represents the log2 fold change of m2AChR activated vs. untreated cells. The y-axis represents the -log 10 of the padj-value, in which 1.3 is equal to a padj of < 0.05. Heatmaps of the top 50 significantly (DEGs) of DPSCs after activation of the m2AChR for 4 h **(C)** and 24 h **(D)** compared to untreated controls. The genes are clustered based on the normalised log2 fold change in gene expression, where the red and green colour scale at the right of the heatmap represents higher and lower relative expression levels, respectively. Each row represents one gene, and each column represents a single sample of the experimental groups. The gene symbols are shown on the right side of the rows. The data is derived from four independent experiments. Statistical analysis was performed using DESeq2 with adjusted p-values calculated by the Benjamini–Hochberg method; genes with padj ≤ 0.05 were considered significantly differentially expressed

Analysis of differentially expressed genes (DEGs) revealed that after 4 h 117 genes were significantly upregulated and 329 significantly downregulated (Fig. 5A). After 24 h stimulation, 205 genes were significantly upregulated and 474 significantly were downregulated (Fig. 5B). Heatmap analysis of normalised log2 fold change in gene expression was utilised to determine the top 50 DEGs (ranked by padj value) at 4 h (Fig. 5C) and 24 h (Fig. 5D). APE treatment resulted predominantly in downregulated gene expression at 4 h, whereas 24 h treatment showed a shift toward upregulation.

**Fig. 6:**
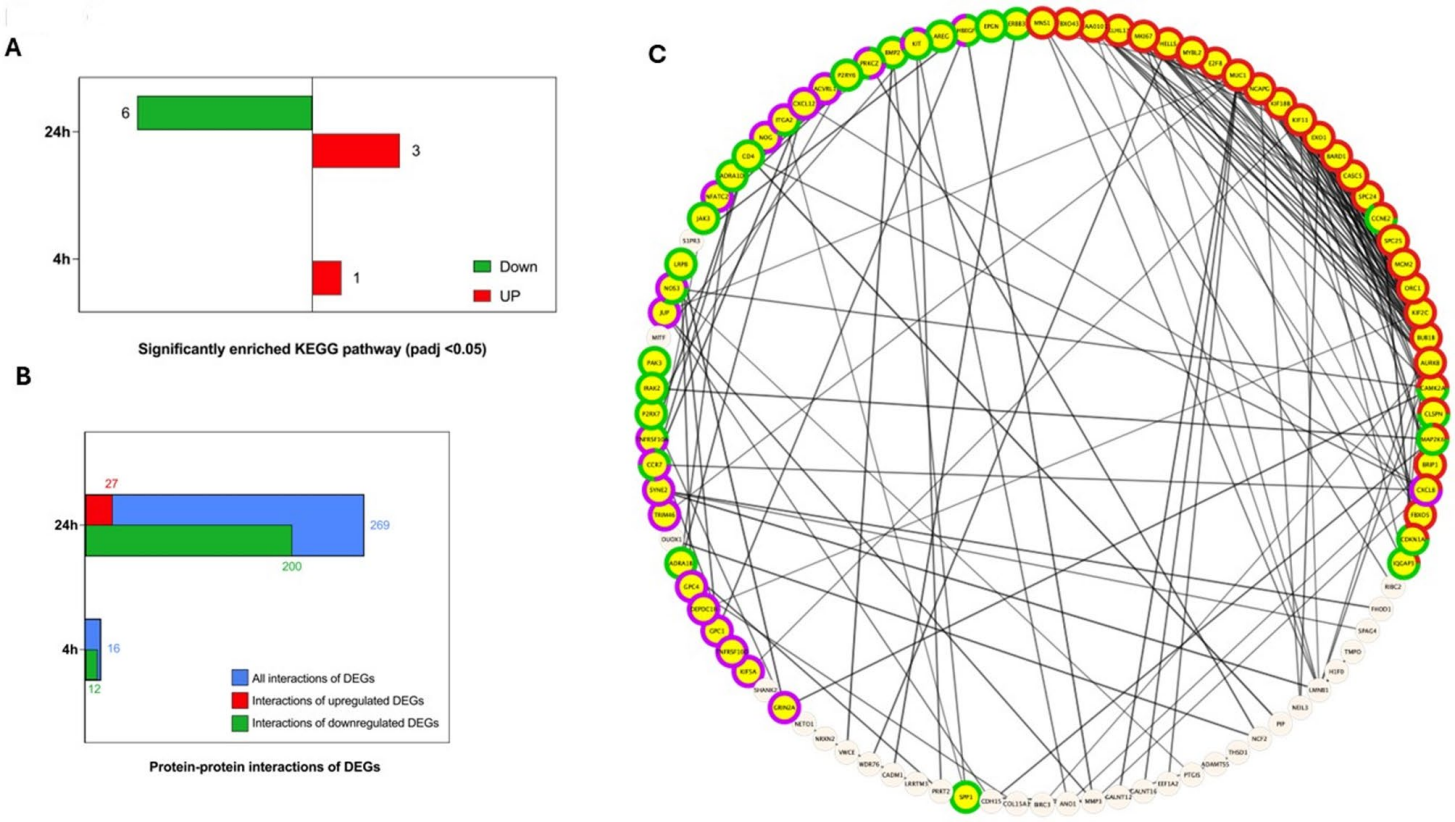
Overview of the biological pathway and protein-protein interaction analysis to determine pathways involved in the m2AChR signalling in dental pulp stem cells. KEGG enrichment analysis of significantly enriched KEGG pathways. **(A)** KEGG enrichment analysis highlighting the number of significantly upregulated and downregulated KEGG pathways after activation of the DPSC m2AChR for 4 and 24 h. **(B)** Protein-protein interaction analysis representing the significant protein coding DEGs after activation of the DPSC m2AChR for 4 and 24 h. **(C)** Interactions between highlighted genes involved in cell cycle (red band), cell migration (purple band), and genes involved in the MAPK cascades (green band)

KEGG pathway enrichment analysis of DEGs revealed that after 4 h incubation with 100 µM APE there was only 1 significantly upregulated KEGG pathway, a metabolic pathway involved in the production and degradation of alanine, aspartate, and glutamate. After 24 h incubation with 100 µM APE there were 6 significantly downregulated KEGG pathways involved in cell metabolism, adhesion, and migration. Moreover, after 24 h incubation with 100 µM APE there were 3 significantly upregulated KEGG pathways involved in cellular metabolism and hormone production (Fig. 6A and supplementary Fig. 6B).

Protein-protein interaction (PPI) analysis was performed based on the STRING database (v11.5) [28], and a PPI network visualization was created with Cytoscape (v3.10.0) [29]. The PPI network of significant DEGs consists of 27 proteins after 4 h m2AChR activation and 184 proteins after 24 h. The proteins at 4 h activation produced 16 pairs of PPIs, while the proteins at 24 h activation produced 269 pairs of PPIs (Fig. 6B).

The PPIs at 4 h activation did not produce enough interactions to generate a meaningful network (data not shown). The main network of PPIs generated from the 24 h m2AChR activation data revealed most interactions were between genes involved in the cell cycle, DNA replication, cell migration and MAPK signalling cascades (Fig. 6C).

### Muscarinic type 2 receptor signalling in dental pulp stem cells engages the MAPK/ERK pathway

The RNAseq data indicated activation of the m2AChR led to upregulation of genes associated with the MAPK/ERK pathway (Fig. 6C). Therefore, subsequent experiments were conducted to determine whether m2AChR activation in DPSCs initiates the MAPK/ERK signalling cascade.

**Fig. 7:**
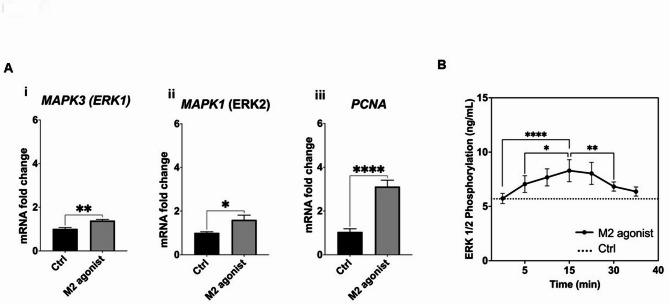
m2AChR signalling in dental pulp stem cells is mediated by the MAPK/ERK signalling pathway. **A** Analysis of expression of genes involved in the MAPK/ERK signalling pathway following stimulation of DPSCs with the M2 agonist (100 µM APE) for 72 h. **(i)** ERK1 (*MAPK3*), **(ii)** ERK2 (*MAPK1*) and **(iii)** Proliferating cell nuclear antigen (*PCNA*), Cells cultured in media alone acted as a control (ctrl). M2 agonist = cells cultured in media containing 100 µM APE. Statistical analysis was performed using unpaired two-tailed t-tests. **B** Time course of phosphorylation of ERK 1/2 after activation of the dental pulp stem cell m2AChR determined using an in-cell ELISA. Cells cultured in media alone acted as a control (ctrl). M2 agonist = cells cultured in media containing 100 µM APE. Statistical analysis was performed using one-way ANOVA with Dunnett’s multiple comparisons test. Data are presented as mean ± SEM from duplicate wells across three independent experiments. *p < 0.05, **p < 0.01, ***p < 0.001, ****p < 0.0001

Targeted qPCR confirmed that expression of key genes involved in MAPK/ERK signalling, including ERK1 (*MAPK3*), ERK2 (*MAPK1*) and Proliferating cell nuclear antigen (*PCNA*), were significantly upregulated (*P* < 0.01, *P* < 0.05 and *P* < 0.0001, respectively) in DPSCs in response to m2AChR activation. Furthermore, an in-cell ELISA confirmed that m2AChR activation caused a transient significant increase in intracellular levels of Phosphorylated ERK1/2 up to 30 min after activation with peak levels being observed after 15 min (*P* < 0.0001, compared to 0 min). Collectively, the data suggests that m2AChR signalling in DPSCs is mediated by the MAPK/ERK pathway Fig. 7.

## Discussion

This study provides the first comprehensive characterisation of the cholinoceptive properties of human DPSCs and supports a model in which m2AChR activation is associated with a reversible quiescent-like state without compromising viability or pluripotency. While DPSCs have been extensively studied for their regenerative and neurogenic potential, their cholinergic signalling pathways have remained unexplored. Here, the expression of muscarinic receptors (mAChRs) and multiple nicotinic receptor (nAChR) subunits was confirmed, and for the first time, the presence of a functional muscarinic type 2 receptor (m2AChR) was demonstrated in DPSCs. Pharmacological activation of this receptor induced a reversible cell cycle arrest, accompanied by suppression of cell migration and osteogenic differentiation, without affecting stemness or viability. These findings suggest that cholinergic pathways, specifically m2AChR signalling, may play a regulatory role in modulating DPSC behaviour and maintaining quiescence.

Previous studies in mesenchymal stem cells (MSCs), with which DPSCs share phenotypic and functional similarities, have suggested the presence of cholinergic signalling components, including AChRs, and implicated cholinergic signalling in the regulation of proliferation and differentiation [30]. In this study, DPSCs were found to express cholinoceptive genes but no transcripts of genes involved in acetylcholine biosynthesis or transport (e.g., *ChAT*, *CrAT*, *VAChT*), indicating that they are not intrinsically cholinergic. However, expression of multiple nAChR subunits, including those that may form functional α7 and α4β2 receptors, were detected, consistent with findings in other MSC populations [14, 29–31]. In addition, expression of the m2, m3, and m5 muscarinic receptors were confirmed at both transcript and protein levels, implicating DPSCs as non-neuronal responders to cholinergic stimuli.

Pharmacological interrogation revealed that m2AChR activation using Arecaidine propargyl ester hydrobromide (APE) significantly inhibited DPSC proliferation. This effect was reversible and not associated with cytotoxicity, apoptosis, or loss of mesenchymal or pluripotency markers, suggesting that the cells were placed into a reversible, non-senescent quiescent state. These observations are consistent with prior studies reporting similar m2AChR-mediated inhibitory effects in other stem cell populations, including rat adipose-derived MSCs and human glioblastoma stem cells [21, 34]. However, this is the first report to show that phenotypically defined DPSCs express a functional m2AChR and that activation of this receptor is associated with a reversible quiescent-like state.

Flow cytometric analysis and gene expression profiling confirmed arrest at the G2/M phase. This was associated with downregulation of mitotic cyclin genes (*CCNA2*, *CCNB1* and *CCNB2*) and upregulation of G1-associated cyclin genes (*CCND1*, *CCND2* and *CCNE2*), *PCNA*, and the CDK inhibitor *CDKN1A* (P21), without a change in *CDKN2A* (P16). The upregulation of P21, a canonical marker of quiescence, alongside unchanged P16 levels, supports the interpretation that the arrest is reversible and not associated with permanent senescence [35, 36]. Increased *PCNA* expression further supports the presence of a transient cell cycle block, rather than loss of replicative potential [37, 38]. Together, these findings suggest that m2AChR activation initiates a P21-driven checkpoint response, placing DPSCs into a reversible quiescent state.

Quiescence, defined as a reversible, non-proliferative state, is conserved across hematopoietic stem cells (HSCs), neural stem cells (NSCs), and mesenchymal stem cells (MSCs), where it serves to protect stem cells from exhaustion and genotoxic damage [39–41]. Beyond p21/p27 and canonical p53/Notch/TGF-β/FOXO axes [40, 42, 43], recent work in human DPSCs has also shown that circadian clock genes (REV-ERBα) can regulate autophagy and inflammation, adding another mechanism by which the function of DPSCs is regulated [44].

In DPSCs, quiescence regulation is less well-characterized. However, our data provide functional evidence suggesting that M2 receptor activation induces a transient and reversible proliferation arrest that is consistent with a quiescent state. Specifically, we observed significant upregulation of CDKN1A (p21), with no change in CDKN2A (p16), which supports a quiescent rather than senescent phenotype. This is consistent with mechanisms reported in MSCs and NSCs, where transient activation of the p53–p21 axis mediates quiescence in response to stress or external signals without triggering permanent arrest [40, 43, 45] Furthermore, the recovery of proliferation following M2 agonist withdrawal, as demonstrated by MTT and CCK8-assays, aligns with the established characteristics of quiescent stem cells re-entering the cell cycle. This mirrors observations in HSC and NSC models, where removal of inhibitory signals leads to renewed proliferation [40, 41].

Quiescence is an important mechanism for preserving stem cell integrity, especially under transient stress or in preparation for differentiation or mobilisation. In this context, the ability to modulate DPSC quiescence via external receptor activation is highly relevant. Importantly, this state was achieved without downregulation of key stemness markers (*CD90*, *CD73* and *CD105*) or pluripotency-associated transcription factors (*Oct4*, *Nanog* and *Sox2*), confirming that m2AChR signalling did not induce spontaneous differentiation but maintained the undifferentiated phenotype (Supplementary Fig. 5, i–vii).

Functionally, activation of the m2AChR impaired both DPSC migration and their ability to undergo osteogenic differentiation. Inhibition of mineralised nodule formation was observed at all induction stages, suggesting that m2AChR activation not only prevents differentiation onset but may also interfere with cells already committed to the osteoblast lineage. Suppression of key osteogenic markers (*ALP*, *BMP2*, *COL1A1* and *SPP1*) further supports this interpretation. The observed impairment in DPSC migration is in line with previous findings in mesenchymal stem cells, where cholinergic signalling modulated cell motility [21]. However, the present data also demonstrate that m2AChR activation directly modulates osteogenic differentiation, as evidenced by phenotypic changes and downregulation of key osteogenic genes. In a broader tissue context, DPSC–endothelial interactions have been demonstrated to be mediated by FN1–ITGA5 and activation of PI3K/AKT signalling promotes endothelial proliferation, migration and tube formation, thereby supporting angiogenesis during pulp development [46]. This process is further reinforced by PDGFRβ^+^ DPSC subpopulations with strong pro-angiogenic activity through PI3K/AKT signalling [47]. These microenvironmental programmes highlight the intrinsic capacity of DPSCs to contribute to vascularisation, providing a benchmark for in vivo behaviour against which receptor-level quiescence may be tuned.

To explore the underlying signalling mechanisms, transcriptomic analysis was performed at 4 and 24 h post-m2AChR activation. Early downregulation of genes involved in metabolism and proliferation was observed, followed by upregulation of stress response and survival pathways. KEGG pathway analysis revealed downregulation of adhesion- and migration-related pathways, many of which were driven by genes encoding cell adhesion molecules (e.g., *CDH15*, *CNTN3*, *PCDHB10*, and *ROBO4*). In contrast, upregulated pathways at 24 h included ferroptosis resistance and steroid biosynthesis, indicating a shift toward metabolic adaptation and survival [48, 49].

Protein–protein interaction analysis revealed that many affected genes clustered around the cell cycle and MAPK signalling. Based on this, and previous reports implicating m2AChR-mediated activation of ERK1/2 in MSCs [13], the role of the MAPK/ERK pathway was investigated. Targeted qPCR and in-cell ELISA confirmed an early, transient upregulation and phosphorylation of ERK1/2 upon APE stimulation, peaking at 15 min and declining by 40 min. While ERK1/2 signalling is classically linked to proliferation, it can also mediate checkpoint restraint depending on activation strength and duration. Studies have shown that sustained ERK1/2 activity can upregulate CDK inhibitors, enforce G2/M arrest, or reactivate APC/C to promote exit into a G0-like state [50–52]. In the present study, the combination of ERK1/2 phosphorylation, enrichment of MAPK-associated genes, and accumulation of DPSCs in G2/M is consistent with checkpoint-mediated quiescence downstream of m2AChR activation. However, further detailed investigations are required to fully delineate the role of the MAPK/ERK pathway in m2AChR signalling using specific pathway inhibitors to fully rule out the role of other signalling cascades such as PI3K/AKT, CXCR7/4, or NRG1/ErbB which may also contribute. In addition, the precise upstream mechanisms linking m2AChR activation to ERK1/2 phosphorylation need to be directly investigated. Previous reports indicate that m2AChRs can signal through Gi/o-mediated inhibition of adenylyl cyclase, through Gβγ subunit release, or via transactivation of receptor tyrosine kinases [13, 22]. In addition, cholinergic control in dental pulp extends beyond muscarinic receptors as evidenced by the fact that the α7nAChR has been shown to regulate autophagy and IL-1β production in deciduous DPSCs, underscoring the need to investigate further the receptor-specific crosstalk between cholinergic pathways and stress-response programs [53].

A further limitation of this study is the absence of direct genetic validation of m2AChR function, such as siRNA knockdown or CRISPR-mediated knockout of *CHRM2*. While our conclusions are supported by selective pharmacological inhibition and downstream functional assays, future studies incorporating gene-specific loss-of-function models will be important to fully confirm the causal role of m2AChR signalling in DPSC quiescence and differentiation. Moreover, as all data were derived from in vitro 2D cultures, it remains uncertain how these findings translate to the native pulp tissue architecture or to complex in vivo microenvironments. Future studies employing 3D pulp-like models or animal systems will be essential to determine the physiological relevance of m2AChR-mediated quiescence and its therapeutic potential in regenerative endodontics.

Taken together, this study indicates that DPSCs possess a functional cholinoceptive system, and that m2AChR activation is associated with engagement of the MAPK/ERK pathway and a reversible quiescent-like state (Supplementary Fig. 7). While the primary findings contribute to fundamental DPSC biology, there are also translational implications worth noting. The ability to pharmacologically induce a reversible, non-toxic quiescent state may be valuable in settings such as in vitro cell banking, storage, or shipment, where maintaining stemness and viability is essential. Recent studies have further demonstrated that PDGFRβ^+^ DPSC subsets remodel extracellular matrix and drive angiogenesis through integrin-mediated pathways, and that FN1–ITGA5–PI3K/AKT signalling facilitates DPSC–endothelial communication during pulp development [46, 47, 54]. Placing reversible m2AChR-mediated quiescence within this broader framework of ECM–integrin regulation highlights how receptor-level control could complement microenvironmental programmes that preserve stemness while supporting vascularisation. Although the clinical application in regenerative endodontics remains speculative and limited by cost, complexity, and current standard-of-care options such as apexification or revascularisation, understanding how to modulate DPSC activity ex vivo could guide future scaffold design or therapeutic preparation protocols. Therefore, further investigation is warranted to assess how such regulatory strategies could integrate with evolving stem cell-based therapies.

## Supplementary Information

Additional file 1.

## Data Availability

The RNAseq data that supports the findings in this manuscript is publicly available in NCBI’s Gene Expression Omnibus and are accessible through GEO Series accession number GSE297021. All remaining raw data and associated materials are freely available upon request from the corresponding author.
