# Supplementary material for: Investigations into the basal neural-like properties of dental pulp stem cells reveal they possess a functional type 2 muscarinic receptor which regulates quiescence
Source: Stem Cell Res Ther. 2025 Nov 12;16:632. doi: 10.1186/s13287-025-04730-7 (PMC12613501; doi:10.1186/s13287-025-04730-7)
Supplement: Supplementary file 1 — Additional file 1. [file 13287_2025_4730_MOESM1_ESM.docx]

**Supplementary Figure 1**

**
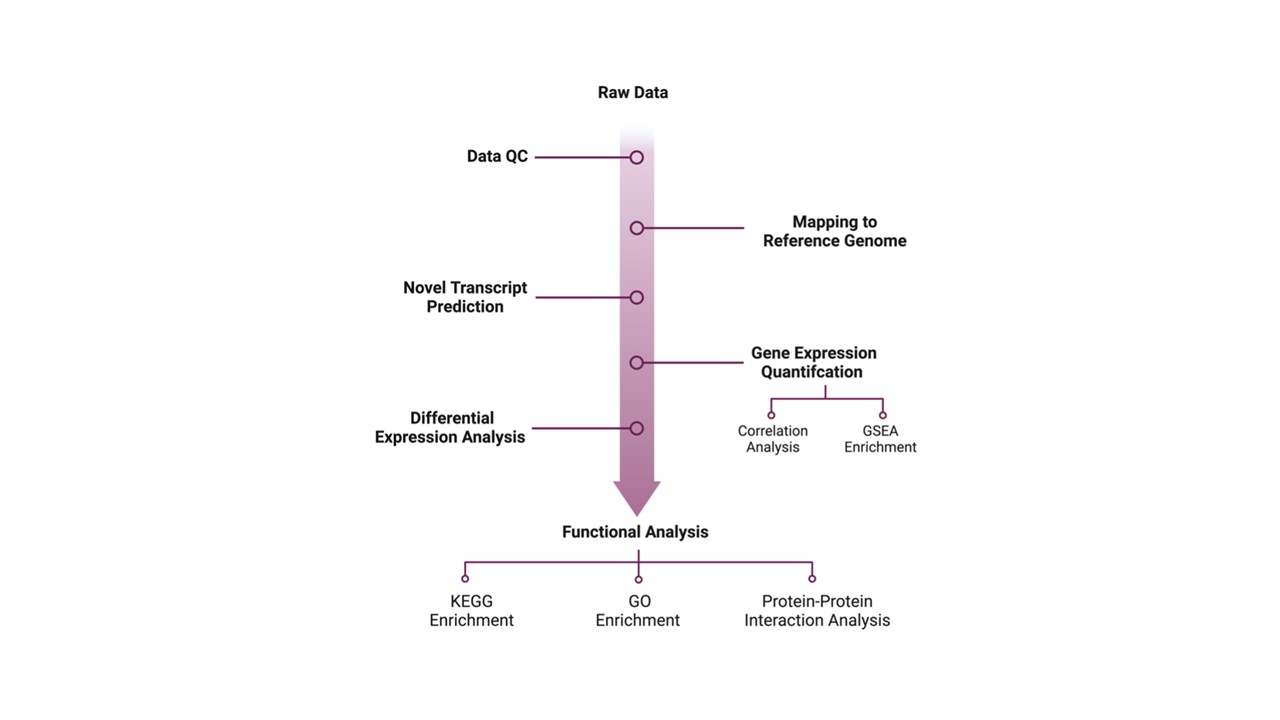
**

**Supplementary Figure 2**

**
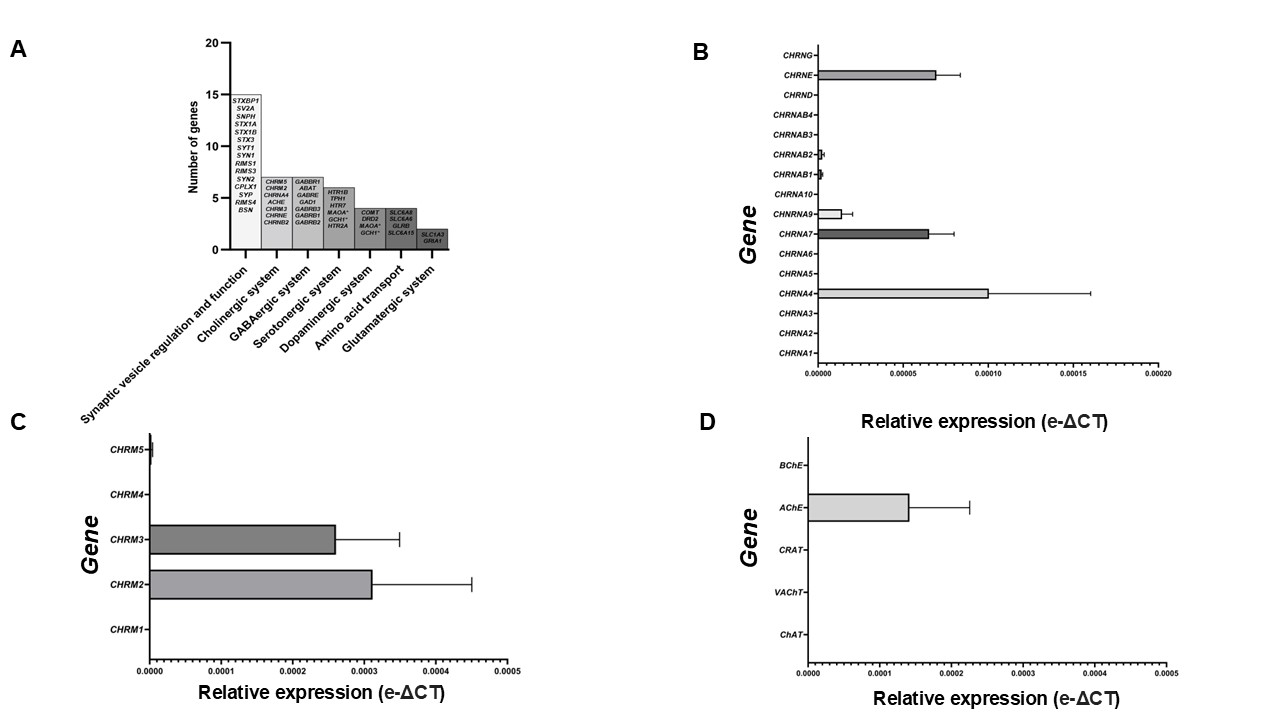
**

**Supplementary Figure 3**

**
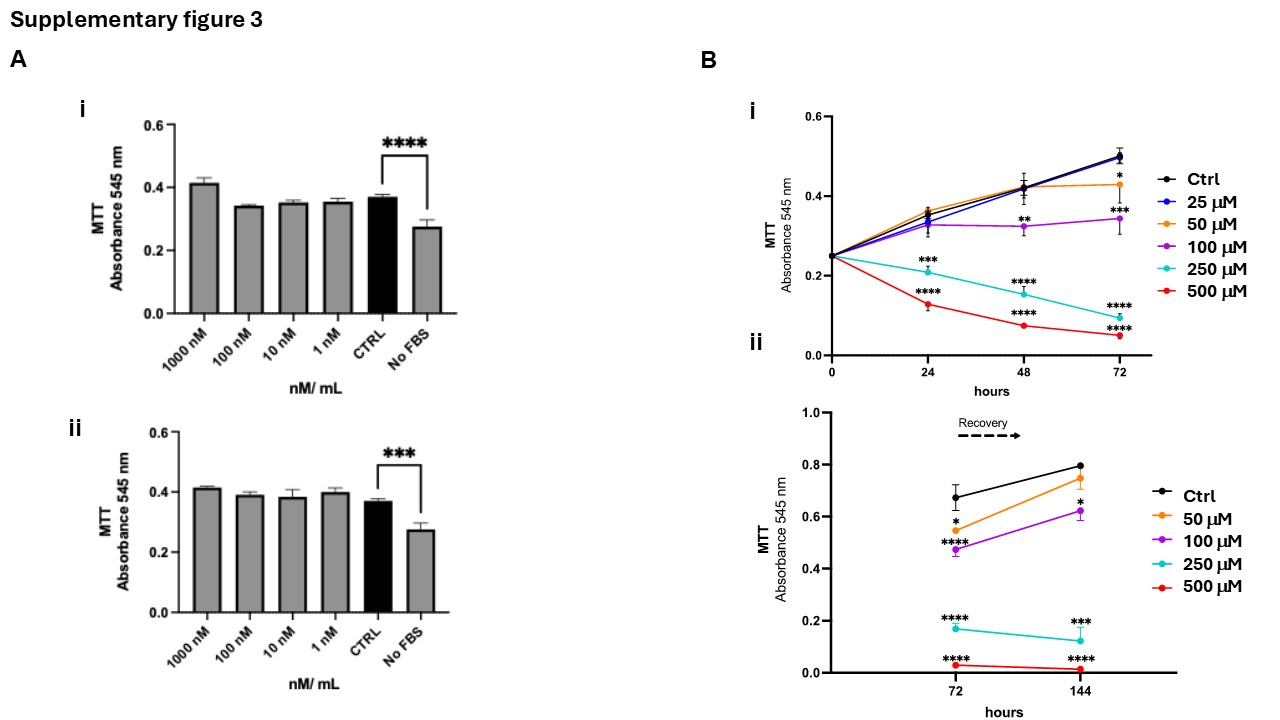
**

**Supplementary Figure 4**

**
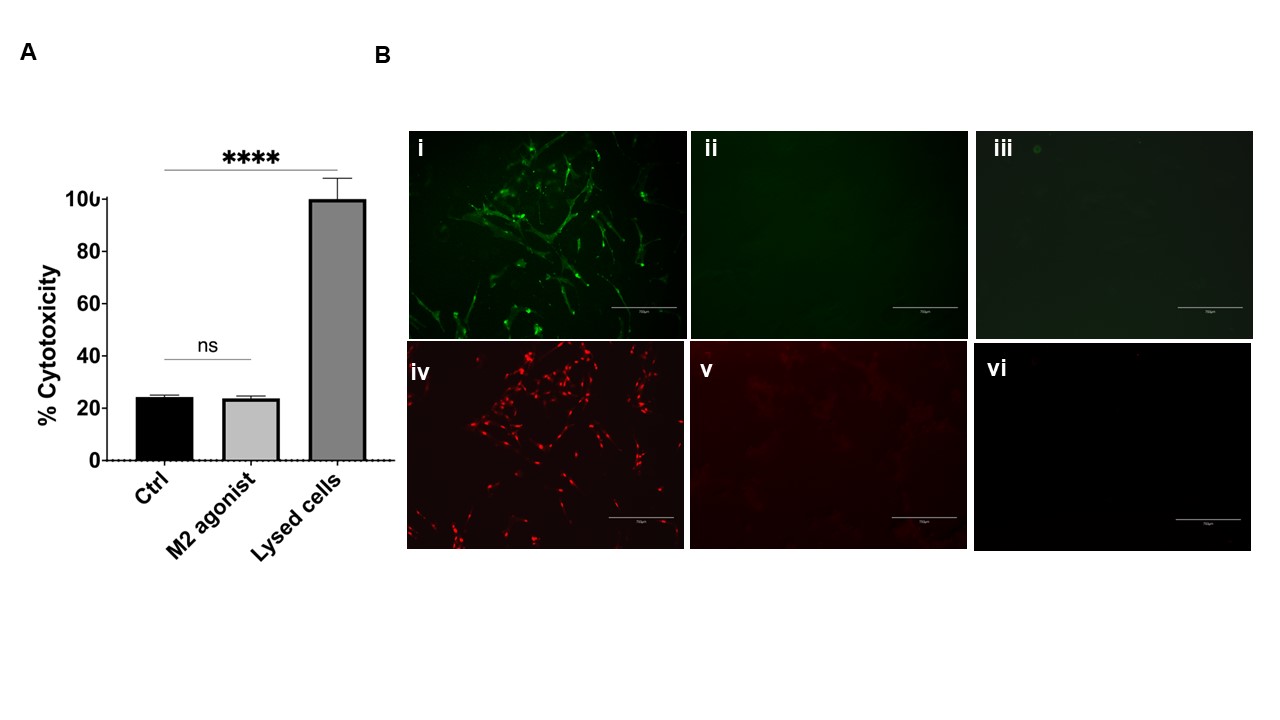
**

**Supplementary Figure 5**

**
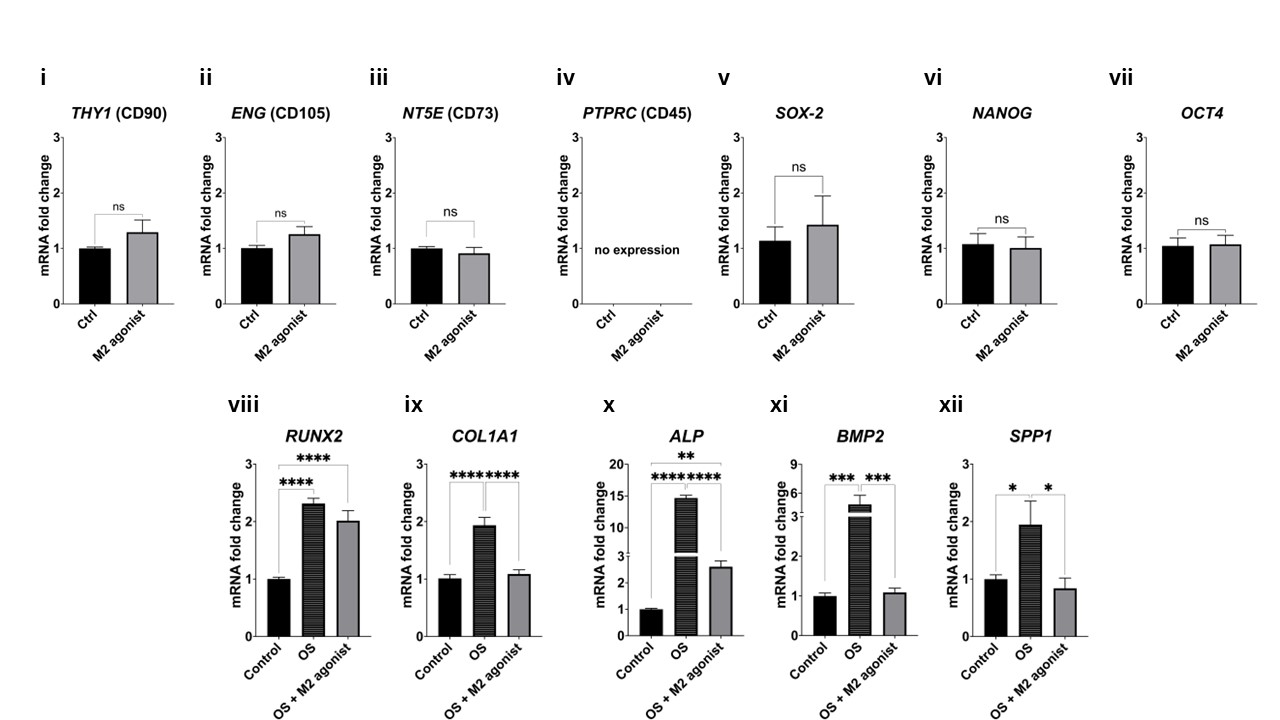
**

**Supplementary Figure 6**

**
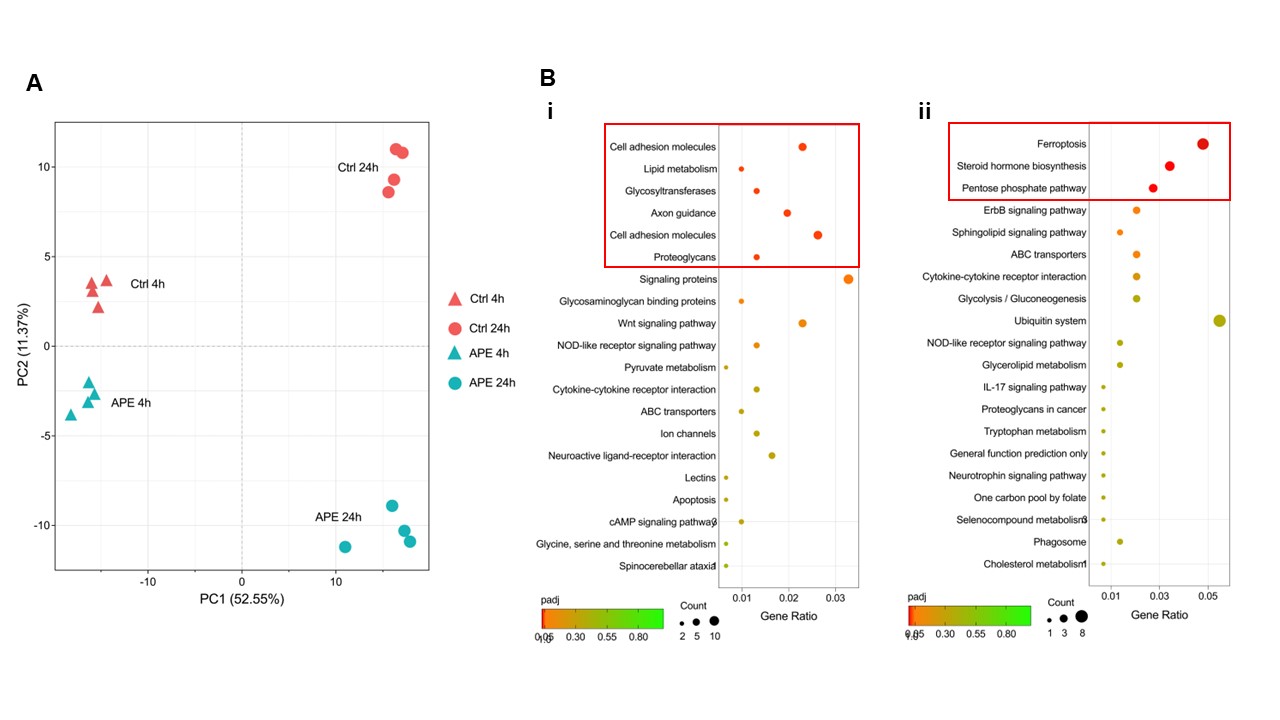
**

**Supplementary Figure 7**


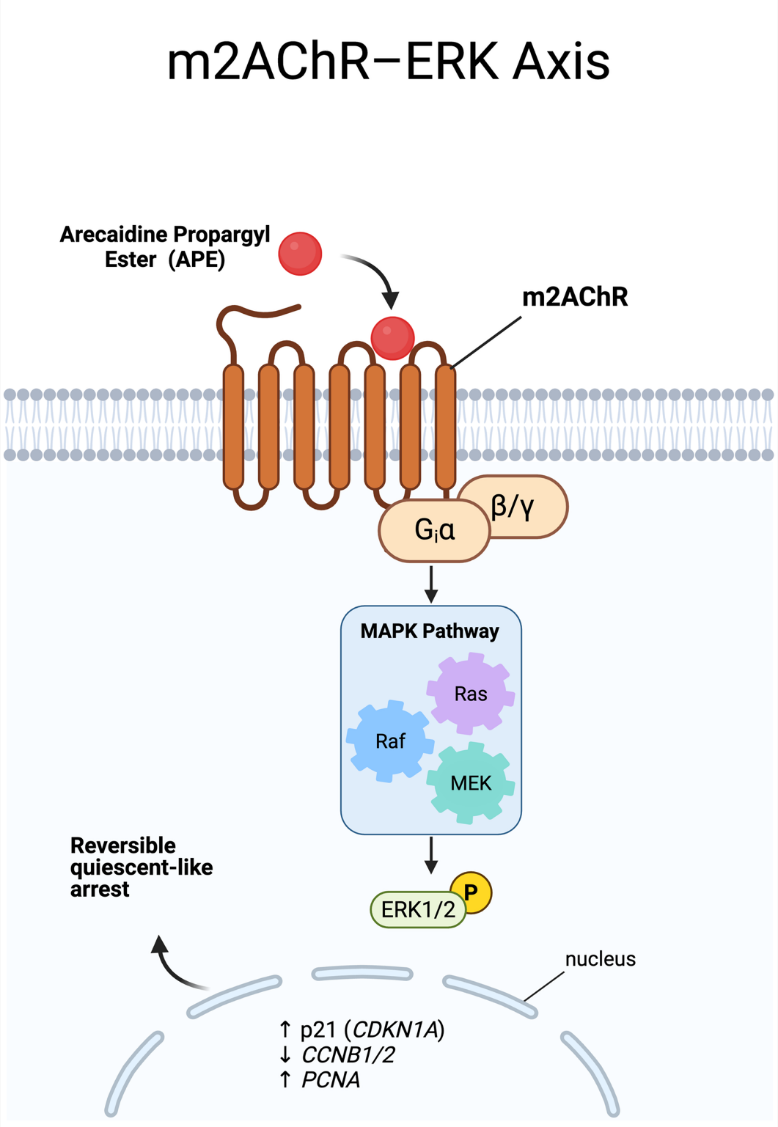


**Figure Legends**

**Figure 1. Analysis of the dental pulp stem cell neurotransmitter profile reveals expression of muscarinic receptors.**

**(A)** Mean gene expression values for the 43/92 neurotransmitter genes determined to be expressed in DPSCs calculated using the e-ΔCT method. The data is derived from six biological replicates (n=6). **(B)** Immunocytochemical analysis of m2AChR. m3AChR and m5AChR expression by DPSCs. **(i)** Expression of the m2AChR protein in DPSCs, **(ii)** Merged images showing DPSCs nuclei stained with DAPI (blue) and actin filaments with phalloidin (green), **(iii)** Merged images of **(i)** and **(ii)**, and **(iv)** Negative control with no primary antibody showing no non-specific binding. **(v)** Expression of the m3AChR protein in DPSCs, **(vi)** Merged images showing DPSCs nuclei stained with DAPI (blue) and actin filaments with phalloidin (green), **(vii)** Merged images of **(v)** and **(vi)**, and **(viii)** Negative control with no primary antibody showing no non-specific binding. **(ix)** Expression of the m5AChR protein in DPSCs, **(x)** Merged images showing DPSCs nuclei stained with DAPI (blue) and actin filaments with phalloidin (green), **(xi)** Merged images of **(ix)** and **(x)**, and **(xii)** Negative control with no primary antibody showing no non-specific binding. All images show 2D projections of confocal stacks and are representative of three independent experiments (n=3). Scale bars = 100 μm.

**Figure 2. Pharmacological induction and inhibition of dental pulp stem cell proliferation confirm the expression of a functional m2AChR**

The effect of the m2AChR agonists **(A)** McN-A 343 (McN, 0.1 – 100 μM) and **(B)** Arecaidine propargyl ester (APE, 0.1 – 100 μM) on DPSC proliferation determined using an MTT assay. The effects on DPSC proliferation after 2 h pretreatment with 100 μM Pirenzepine (Pz) and 0.1 μM Methoctramine (MC), alone or in combination, followed by 72 h treatment with **(C)** 100 μM McN and **(D)** 100 μM APE. Comparison made to untreated group (CTRL); negative controls are cells without serum (No FBS). **(E)** Time course of DPSC proliferation after stimulation with 100 mM APE. Ctrl denotes DPSCs cultured in media alone, 100 μM denotes DPSCs cultured in media containing 100 μM APE and recovery denotes DPSCs cultured in media containing 100 μM APE for 72 h and then replacing with culture media alone. **(F)** CCK-8 assessment of live cells over a 120 h period. m2AChR agonist depicts viable cells after stimulation with 100 μM APE. Serum starved cells (NO FBS) acted as a negative control and cells in media alone containing serum (Ctrl) acted as a positive control. Data are presented as mean ± SEM from duplicate wells across three independent experiments. Statistical analysis was performed using one-way ANOVA; Dunnett’s multiple comparisons test was used for panels A–D, and Tukey’s post hoc test was used for panels E and F. *p < 0.05, **p < 0.01, ***p < 0.001, ****p < 0.0001. Full dose–response and recovery data for APE are shown in Supplementary Fig. 3B, and antagonist-alone controls are provided in Supplementary Fig. 3A.

**Figure 3. Activation of the m2AChR arrests dental pulp stem cell proliferation at the G2/M phase**

**(A)** Cell cycle analysis of DPSCs after 72 h of stimulation with the M2 agonist (100 mM APE) using bivariate analysis of BrdU incorporation (ordinate) and propidium iodide (PI) labelled DNA content (abscissa). **(i)** Example bivariate plot of DPSCs cultured in media alone (ctrl) and gating strategy. **(ii)** Example bivariate plot of DPSCs cultured in media containing 100 μM APE (M2 agonist) and gating strategy. **(iii)** Graphical representation of cell cycle analysis. **(B)** Analysis of expression of genes involved in proliferation and the cell cycle following stimulation with the M2 agonist (100 μM APE) for 72 h. **(i)** cyclin-A2 (*CCNA2*), **(ii)** cyclin- B1 (*CCNB1*), **(iii)** cyclin-D1(*CCND1*), **(iv)** cyclin-D2 (*CCND2*), **(v)** cyclin-E2 (*CCNE2*), **(vi)** cell division cycle 14A (*CDC14A*), **(vii)** M phase inducer phosphatase 1 (*CDC25A*), **(viii)** cyclin dependent kinase inhibitor 1A (*CDKN1A*), **(ix)** glycogen synthase kinase 3 beta (*GSK3B*) and **(x)** proliferating cell nuclear (*PCNA*). Cells cultured in media (undifferentiated) alone acted as a control (ctrl). M2 agonist = cells cultured in media containing 100 μM APE. Data are presented as mean ± SEM from duplicate wells across three independent experiments. Statistical analysis was performed using unpaired two-tailed t-tests for all comparisons. *p < 0.05, **p < 0.01, ***p < 0.001, ****p < 0.0001.

**Figure 4. Activation of the dental pulp stem cell m2AChR inhibits their migration and osteogenic differentiation**

**(A)** Determination of the effect of m2AChR activation on DPSC migration using a scratch assay. **(i)** DPSCs grown on glass coverslips in control media, exposed to a scratch and stained for actin (green) after 8 h. **(ii)** DPSCs grown on glass coverslips in media containing 100 μM APE, exposed to a scratch and stained for actin (green) after 8 h. **(iii)** DPSCs grown on glass coverslips in media containing 0.01 μM MC (m2AChR antagonist) and 100 μM APE, exposed to a scratch and stained for actin (green) after 8 h. Images are representative of duplicate coverslips from three independent experiments. **(iv)** Quantification of DPSC migration measured by determining the difference in the width of the gap (as shown by yellow arrow) at 0 h and 8 h. Statistical analysis was performed using one-way ANOVA with Tukey’s post hoc test. Data are presented as mean ± SEM (n = 3). ns: not significant, ****p < 0.0001. Scale bars = 1200 μm. APE= Arecaidine propargyl ester, MC= Methoctramine. **(B)** Determination of the effect of m2AChR activation on DPSC osteogenic differentiation. DPSCs grown on glass coverslips for 4 weeks in normal media and stained with Alazarin red. **(ii)** DPSCs grown on glass coverslips for 4 weeks in osteogenic differentiation media and stained with Alazarin red. **(iii)** DPSCs grown on glass coverslips for 4 weeks in osteogenic differentiation media containing 100 μM APE and stained with Alazarin red. **(iv)** DPSCs grown on glass coverslips for 4 weeks in normal media and stained with von Kossa. **(v)** DPSCs grown on glass coverslips for 4 weeks in osteogenic differentiation media and stained with von Kossa. **(vi)** DPSCs grown on glass coverslips for 4 weeks in osteogenic differentiation media containing 100 μM APE and stained with von Kossa. Images are representative of duplicate coverslips from three independent experiments. **(vii)** Alizarin Red stain quantification showing significantly less mineralisation compared to cells that had undergone osteogenic differentiation. Statistical analysis was performed using one-way ANOVA with Kruskal–Wallis post hoc test. Data are presented as mean ± SEM (n = 3). *p < 0.05, ****p < 0.0001.

**Figure 5. Overview of differential gene expression by dental pulp stem cells after activation of the m2AChR.**

Volcano plots for differentially expressed genes (DEGs) of DPSCs after activation of the m2AChR for 4 h **(A)** and 24 h **(B)** compared to untreated controls. Scatter points represent genes. The x-axis represents the log 2 fold change of m2AChR activated vs untreated cells. The y-axis represents the -log 10 of the padj-value, in which 1.3 is equal to a padj of <0.05. Heatmaps of the top 50 significantly (DEGs) of DPSCs after activation of the m2AChR for 4 h **(C)** and 24 h **(D)** compared to untreated controls. The genes are clustered based on the normalised Log2 fold change in gene expression, where the red and green colour scale at the right of the heatmap represents higher and lower relative expression levels, respectively. Each row represents one gene, and each column represents a single sample of the experimental groups. The gene symbols are shown on the right side of the rows. The data is derived from four independent experiments. Statistical analysis was performed using DESeq2 with adjusted p-values calculated by the Benjamini–Hochberg method; genes with padj ≤ 0.05 were considered significantly differentially expressed.

**Figure 6. Overview of the biological pathway and protein-protein interaction analysis to determine pathways involved in the m2AChR signalling in dental pulp stem cells.**

KEGG enrichment analysis of significant enriched KEGG pathways. **(A)** KEGG enrichment analysis highlighting the number of significantly upregulated and downregulated KEGG pathways after activation of the DPSC m2AChR for 4 and 24 h. **(B)** Protein-protein interaction analysis representing the significant protein coding DEGs after activation of the DPSC m2AChR for 4 and 24 h. **(C)** Interactions between highlighted genes involved in cell cycle (red band), cell migration (purple band), and genes involved in the MAPK cascades (green band).

**Figure 7. m2AChR signalling in dental pulp stem cells is mediated by the MAPK/ERK signalling pathway**

**(A)** Analysis of expression of genes involved in the MAPK/ERK signalling pathway following stimulation of DPSCs with the M2 agonist (100 μM APE) for 72 h. **(i)** ERK1 (*MAPK3*), **(ii)** ERK2 (*MAPK1*) and **(iii)** Proliferating cell nuclear antigen (*PCNA*)*,* Cells cultured in media alone acted as a control (ctrl). M2 agonist = cells cultured in media containing 100 μM APE. Statistical analysis was performed using unpaired two-tailed t-tests. **(B)** Time course of phosphorylation of ERK 1/2 after activation of the dental pulp stem cell m2AChR determined using an in-cell ELISA. Cells cultured in media alone acted as a control (ctrl). M2 agonist = cells cultured in media containing 100 μM APE. Statistical analysis was performed using one-way ANOVA with Dunnett’s multiple comparisons test. Data are presented as mean ± SEM from duplicate wells across three independent experiments. *p < 0.05, **p < 0.01, ***p < 0.001, ****p < 0.0001.

**Supplementary Figure 1. Bioinformatic analysis pipeline**

The schematic diagram shows the bioinformatics pipeline used by Novogene for data analysis. Raw sequences were subjected to a quality control checkpoint to achieve high quality reads. Reads were mapped to the human reference genome, and the aligned transcripts were assembled and quantified. Differentially expressed genes were analysed for significant pathways enrichment using different databases.

**Supplementary Figure 2. Cholinergic and cholinoceptive gene expression in dental pulp stem cells.**

**(A)** Graphical representation of the expressed neurotransmitter genes grouped by function. List within bars is ordered by mean gene expression values. The data is derived from six biological replicates (n=6). *Genes involved in the synthesis of both serotonin and dopamine. **(B)** Targeted qPCR analysis of nicotinic receptor (nAChR) subunit gene expression in dental pulp stem cells. **(C)** Targeted qPCR analysis of muscarinic receptor (mAChR) gene expression in dental pulp stem cells. **(D)** Targeted qPCR analysis of key enzyme and transporter gene expression for the synthesis and metabolism of acetylcholine (ACh) in dental pulp stem cells. In all panels the data is derived from duplicate wells of three independent experiments (n=3) and the relative abundance of each transcript calculated using the e-ΔCT method. *CHRM1* – *CHRM5* = Muscarinic receptor 1 – 5, *CHRNA1* – *CHRNA7, CHRNA9, CHRNA10*  = nicotinic receptor subunits a1 – a7, a9 and a10, *CHRNB1* – *CHRNB4* = nicotinic receptor subunits b1 – b4, *CHRND* = nicotinic receptor subunit d, *CHRNE* = nicotinic receptor subunit e, *CHRNG* = nicotinic receptor subunit g, ACHE = Acetylcholinesterase, BCHE = Butyrylcholinesterase, CHAT = Choline acetyltransferase, CRAT = Carnitine acyltransferase, VAChT = Vesicular acetylcholine transporter, ND = no expression determined. ** p< 0.01, **** p<0.0001.

**Supplementary Figure 3. Does response inhibition of dental pulp stem cell proliferation using the m2AChR agonist, Arecaidine propargyl ester, and pharmacological competition assays to show m2AChR specificity.**

**(A)** Dose response inhibition and recovery of DPSC proliferation after stimulation with varying concentrations of the m2AChR agonist (APE). **(i)** Dose response inhibition of DPSCs proliferation using 0 – 500 μM APE. Ctrl = 0 μM APE (untreated). **(ii)** Proliferative recovery of DPSCs after stimulation with 0 – 500 μM APE for 72 h and removal of APE for a further 72 h. Data is derived from duplicate wells of three independent experiments. Statistical analysis was performed using a one-way ANOVA and Dunnett’s multiple comparisons test *p < 0.05, **p < 0.01, ***p < 0.001, ****p < 0.0001. Concentrations of APE ≥250 μM caused irreversible changes in cell proliferation suggestive of cellular toxicity. **(B)** The effects of **(i)** 1 – 1000 nm Pirenzepine (PZ) and **(ii)** 1 – 1000 nm Methoctramine (MC) alone on DPSCs proliferation. Comparison made to untreated group (CTRL); negative control are cells without serum (No FBS). All Data was derived from duplicate wells of three independent experiments. Statistical analysis was performed using a one-way ANOVA and Dunnett’s multiple comparisons test *p < 0.05, **p < 0.01, ***p < 0.001, ****p < 0.0001. None of the concentrations of the compounds used in this study significantly affected DPSCs proliferation.

**Supplementary Figure 4. Activation of the dental pulp stem cell m2AChR with the specific agonist APE does not cause cell necrosis or apoptosis.**

**(A)** LDH release from DPSCs after activation of the m2AChR for 72 h with 100 μM APE. Ctrl shows DPSCs cultured in media alone (negative control) and lysed cells shows cells cultured in media alone followed by lysis with a commercial lysis buffer (positive control). Data is derived from duplicate wells of three independent experiments (n=3). **(B)** Fluorescent microscopy images of Annexin V/PI stained cells. **(i)** Annexin V staining of DPSCs after exposure to 30% methanol to induce apoptosis. **(ii)** Absence of Annexin V staining in the untreated control (DPSCs in media alone) and **(iii)** DPSC m2AChR activation with 100 μM APE. **(iv)** PI staining of DPSCs after exposure to 70% methanol to induce necrosis. **(v)** Absence of PI staining in the untreated control (DPSCs in media alone) and **(vi)** DPSC m2AChR activation with 100 μM APE. All images show 2D projections of confocal stacks and are a representative of three independent experiments (n=3). Scale bars = 750 μm.

**Supplementary Figure 5. Activation of the m2AChR has no effect on dental pulp stem cell stemness or pluripotency but inhibits their osteogenic differentiation**

Analysis of expression of genes identified as markers of stemness and pluripotency following stimulation of DPSCs with the M2 agonist (100 μM APE) for 72 h. **(i)** CD90 (*THY1*), **(ii)** CD105 (*ENG*), **(iii)** CD73 (*NTSE*), **(iv)** *CD45 (PTPRC)*, **(v)** SRY-Box Transcription Factor 2 (*SOX-2*), **(vi)** Homeobox Transcription Factor Nanog (*NANOG*) and **(vii)** OCT4 (*POU5F1*). Cells cultured in media (undifferentiated) alone acted as a control (ctrl). M2 agonist = cells cultured in media containing 100 μM APE. Analysis of expression of key genes involved in the DPSC osteogenic differentiation process as determined by qPCR: **(viii)** *RUNX2***, (ix)** *COL1A1* **(x)** *ALP* **(xi)** *BMP2* **(xii)** *SPP1*. Cells cultured in normal media (undifferentiated) acted as a control. OS = cells cultured in osteogenic differentiation media, OS +M2 agonist = cells cultured in osteogenic differentiation media containing 100 μM APE. All data is derived from duplicate wells of three independent experiments. * p< 0.05, ** p< 0.01, *** p<0.001, **** p<0.0001.

**Supplementary Figure 6. Principal Component Analysis (PCA) and KEGG pathway analysis**

**(A)** Grouping of samples was based on variance in expression. PCA analysis of differential gene expression in response to M2 activation via APE and the untreated controls (Ctrl). PC1 shows the largest variance (52%) along the x-axis and PC2 displays the second largest variance (11%) on the y-axis. **(B)** Top 20 downregulated and upregulated KEGG pathways. **(i)** Shows the top 20 significantly downregulated KEGG pathways (ranked by p-value), with the significant pathways highlighted in the red box). **(ii)** Shows the top 20 significantly upregulated KEGG pathways (ranked by p-value), with the significant pathways highlighted in the red box). Gene ratio (x-axis) is the percentage of significant genes over the total genes in each pathway. The size of the dot is based on gene count enriched in the pathway.

**Supplementary Figure 7. Proposed pathway by which activation of the m2AChR promotes reversible quiescent-like arrest in human DPSCs**

Activation of the m2AChR, by the specific agonist APE, causes Gi/o-mediated inhibition of adenylyl cyclase or Gβγ subunit release which in turn activates the MAPK/extracellular signal-regulated kinase (ERK) pathway causing ERK1/2 phosphorylation which mediates checkpoint restraint.
